# NDFIP2 Suppresses Colorectal Cancer Metastasis Through DDX3X‐Dependent R‐Loop Remodeling and GNG13 Transcriptional Activation

**DOI:** 10.1002/advs.78466

**Published:** 2026-10-31

**Authors:** Enjian Zhang, Yanfang Li, Jiaxin Shu, Shang Xu, Heng Lv, Chong Zhang, Pengbo Zhang, Nai Wu, Shuai Gong, Xiuzhong Zhang, Zeqiang Ren, Yi Zhang, Xin Liu

**Affiliations:** ^1^ Department of Gastrointestinal Surgery Affiliated Hospital of Xuzhou Medical University Xuzhou China; ^2^ Jiangsu Key Laboratory of Organ Transplantation and Transplant Immunology Xuzhou Medical University Xuzhou China; ^3^ Technology Innovation and Public Service Center Huaihai Medical Science and Technology Innovation Center The Affiliated Hospital of Xuzhou Medical University Xuzhou China; ^4^ Department of Endocrinology Affiliated Hospital of Xuzhou Medical University Xuzhou China; ^5^ Department of Biology and Biochemistry Center For Nuclear Receptors and Cell Signaling University of Houston Houston Texas USA; ^6^ Department of Epigenetics and Molecular Carcinogenesis University of Texas MD Anderson Cancer Center Houston Texas USA

**Keywords:** colorectal cancer, DDX3X, iRGD, metastasis, NDFIP2, PI3K/AKT signaling, R‐loop

## Abstract

Distant metastasis remains the leading cause of colorectal cancer (CRC) mortality, yet the underlying molecular mechanisms remain incompletely defined. Integrating RNA sequencing (RNA‐seq) profiling and clinical validation, we identified NDFIP2 as significantly downregulated in metastatic CRC. Functionally, NDFIP2 loss drove epithelial‐mesenchymal transition (EMT), stemness, and metastasis via PI3K/AKT hyperactivation. Mechanistically, NDFIP2 serves as an adaptor recruiting the E3 ligase NEDD4 to induce K48‐linked polyubiquitination and proteasomal degradation of DDX3X. Unrestrained DDX3X resolves R‐loops at the GNG13 promoter and recruits the transcription factor CTCF, synergistically enhancing GNG13 transcription to amplify PI3K/AKT signaling. Targeting this vulnerability, we engineered a cell‐permeable NDFIP2‐derived iRGD‐conjugated peptide (ND‐iRGD). In vivo administration of this construct suppressed DDX3X/GNG13/PI3K/AKT signaling, thereby attenuating distant metastasis and prolonging murine survival. Collectively, NDFIP2 acts as a critical metastasis suppressor via the DDX3X/R‐loop/GNG13 axis, presenting the ND‐iRGD peptide as a promising translatable therapeutic strategy for metastatic CRC.

## Graphical abstract

NDFIP2 recruits NEDD4 to promote K48‐linked ubiquitination and proteasomal degradation of DDX3X, leading to R‐loop accumulation and CTCF‐dependent suppression of GNG13 transcription. Consequently, PI3K/AKT signaling, EMT, stemness, and colorectal cancer metastasis are inhibited. An iRGD‐NDFIP2 fusion peptide recapitulates this metastasis‐suppressive effect, highlighting its potential therapeutic value.

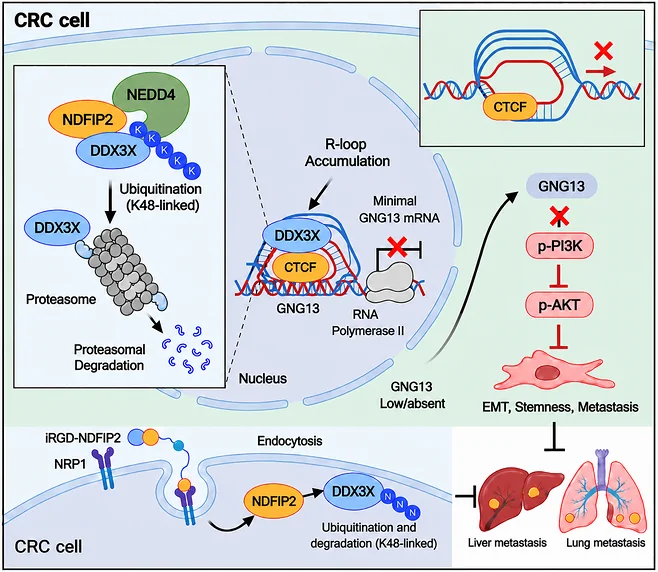

**Abbreviations**
BSA: Bovine serum albumin
CAFs: Cancer‐associated fibroblasts
Co‐IP: Co‐immunoprecipitation
CQ: Chloroquine
CRC: Colorectal cancer
EMT: Epithelial‐mesenchymal transition
FBS: Fetal bovine serum
gDNA: Genomic DNA
H&E: Hematoxylin and eosin
HCC: Hepatocellular carcinoma
IP: Immunoprecipitation
KO: Knockout
OE: Overexpression
ORR: Objective response rate
p‐AKT: Phospho‐AKT
p‐PI3K: Phospho‐PI3K
PFS: Progression‐free survival
PPII: Polyproline type II
RNAPII: RNA polymerase II
scRNA‐seq: Single‐cell RNA‐sequencing
TME: Tumor microenvironment
TNBC: Triple‐negative breast cancer
WT: Wild‐type

## 1 Introduction

Colorectal cancer (CRC) is among the most common malignancies worldwide, with distant metastasis representing the major cause of mortality [1]. Approximately 20%–25% of patients present with distant metastasis at diagnosis, and 30%‐40% of those with localized disease eventually develop metastatic progression [2]. However, current therapies for metastatic CRC remain largely ineffective, highlighting the urgent need to elucidate the mechanisms underlying CRC metastasis and to develop more effective therapeutic strategies [3].

NEDD4 family interacting protein 2 (NDFIP2) is a NEDD4‐interacting protein that recruits NEDD4 and facilitates its engagement with specific substrates, thereby regulating substrate ubiquitination efficiency. NDFIP2 plays an important role in the regulation of iron metabolism and hepatic homeostasis. For instance, NDFIP2 maintains hepatic iron homeostasis by regulating the iron transporter DMT1 [4]. However, the role of NDFIP2 in cancer has been only rarely reported, and recent research has developed a 9‐gene predictive model, which includes NDFIP2, that demonstrates good efficacy in predicting peritoneal metastasis in gastric cancer [5]. This indicates that NDFIP2 might exert a crucial regulatory function in gastrointestinal malignancies. Nevertheless, the exact functions and mechanisms remain to be elucidated.

NEDD4 is a HECT‐type E3 ubiquitin ligase that influences tumor progression by regulating the ubiquitination and stability of its substrates. Aberrant NEDD4 expression or activity has been implicated in the progression of multiple malignancies, including colorectal, breast, lung, gastric, and prostate cancers [6]. DDX3X is an ATP‐dependent DEAD‐box RNA helicase involved in transcription, RNA splicing, nuclear export, and translation, and its dysregulation has been linked to tumor growth, stemness, and metastasis in multiple cancers [7].

R‐loops are dynamic RNA:DNA hybrid structures that are widely formed during transcription and participate in multiple physiological processes, including gene regulation and genome maintenance [8]. However, dysregulated R‐loop homeostasis can lead to their excessive accumulation, particularly at promoter regions, thereby perturbing gene transcription, compromising genome stability, and potentially promoting tumor progression [9]. Previous studies have demonstrated that the aberrant accumulation of R‐loops at the promoter of the tumor suppressor BDH1 recruits HDAC2 and CHD4 to assemble a transcriptional repressor complex. This complex induces chromatin compaction to silence BDH1 transcription, thereby driving hepatocellular carcinoma (HCC) progression [10]. Additionally, R‐loops can facilitate the nuclear translocation of β‐catenin to promote tumor stemness in triple‐negative breast cancer (TNBC) [11]. Collectively, R‐loops exert transcriptional regulatory effects at gene promoter regions and can directly or indirectly influence the activation of tumor‐related pathways, thereby modulating tumor progression.

In the present study, utilizing multi‐omics sequencing combined with online public databases, we identified NDFIP2 as a suppressor of CRC metastasis. Both in vivo and in vitro experiments confirmed the suppressive effect of NDFIP2 on CRC metastasis. Mechanistic investigations revealed that NDFIP2 mediates the ubiquitin‐dependent degradation of DDX3X [12]. DDX3X, in turn, acts as a transcriptional activator of GNG13 by resolving R‐loops at its promoter [13] and aiding CTCF recruitment, thereby triggering the PI3K/AKT pathway. Consequently, NDFIP2 disrupts this DDX3X/GNG13 axis to effectively inhibit PI3K/AKT signaling and attenuate colorectal cancer metastasis. Furthermore, an engineered ND‐iRGD fusion peptide exhibited potent anti‐metastatic activity in both in vitro and in vivo models. These findings suggest that NDFIP2 may serve as a potential therapeutic target for CRC metastasis.

## 2 Materials and Methods

### 2.1 Patient Specimens

Surgically resected primary tumor tissues and matched adjacent non‐tumor tissues were collected from six CRC patients with liver metastasis and six CRC patients without distant metastasis at the Affiliated Hospital of Xuzhou Medical University. An independent cohort comprising three T2N0M0 and three T2N0M1 tumors was used for bulk RNA sequencing, whereas a separate independent cohort comprising three T2N0M0 and three T2N0M1 tumors was used for single‐cell RNA sequencing. For bulk RNA‐seq, tissue specimens were snap‐frozen in liquid nitrogen immediately after resection and subsequently processed for RNA extraction and sequencing. Fresh tumor specimens were processed for single‐cell RNA sequencing (scRNA‐seq) as appropriate. The study was reviewed and approved by the Ethics Committee of the Affiliated Hospital of Xuzhou Medical University (approval no. XYFY2025‐KL386‐01), and all procedures involving human participants were conducted in accordance with the Declaration of Helsinki. Written informed consent was obtained from all participants prior to surgery.

### 2.2 Cell Culture, Plasmid Transfection, and Virus Infection

Human CRC cell lines (HCT116, LoVo) and the human embryonic kidney 293T cell line were purchased from Procell. All cell lines were recently authenticated using Short Tandem Repeat (STR) profiling (RRID: CVCL_0291 for HCT116; RRID: CVCL_0399 for LoVo; RRID: CVCL_0063 for HEK293T) and were routinely tested for mycoplasma contamination using polymerase chain reaction (PCR)‐based kits every three months. HCT116 and 293T cells were cultured in DMEM (Gibco, 11965‐092) supplemented with 10% fetal bovine serum (FBS; ADSbio, ADS‐FBS701), whereas LoVo cells were maintained in RPMI 1640 (Gibco, 11875093) containing 10% FBS. For plasmid and small interfering RNA (siRNA) transfection, Lipofectamine 3000 and P3000 reagents (Invitrogen, L3000008) were used according to the manufacturer's instructions. To establish stably transfected cell lines, HCT116 and LoVo cells were infected with lentivirus in serum‐free medium. Twelve hours after infection, the medium was replaced with complete medium. At 48 h post‐infection, puromycin (MedChemExpress, HY‐B1743) was added at 1 µg/mL for selection for 3 days, after which the concentration was increased to 1.5 µg/mL for an additional 3 days. On day 8 after infection, the efficiency of the indicated genetic manipulation was confirmed by western blotting.

### 2.3 Plasmid Construction

The pLKO.1 vector was used for short hairpin RNA (shRNA)‐mediated gene silencing, pcDNA3.1 for gene overexpression, and lentiCRISPR v2 for CRISPR/Cas9‐mediated gene knockout. Plasmids encoding HA‐tagged wild‐type ubiquitin and the K48R and K63R ubiquitin mutants were purchased from YouBio (Changsha, China). The target sequences of the shRNAs, single‐guide RNAs (sgRNAs), and siRNAs used in this study are listed in Supplementary Material.

### 2.4 Cell Migration and Invasion Assays

Adherent CRC cells were digested with 0.25% trypsin (Beyotime Biotechnology, C0201‐100 mL). After digestion, serum‐containing medium was added to neutralize trypsin, and the cells were resuspended. Cell concentration was determined using a cell counter. For the Transwell migration assay, 200 µL of serum‐free medium containing approximately 2 × 10^4^ cells was added to the upper chamber of a Transwell insert with an 8 µm pore size (JET BIOFIL, TCS001024), while 700 µL of serum‐containing medium was added to the lower chamber. After incubation for 48 h, the non‐migrated cells on the upper surface of the membrane were gently removed with a cotton swab. The migrated cells were then fixed with 4% paraformaldehyde (Biosharp, BL539A) for 20 min and stained with 0.1% crystal violet (Beyotime Biotechnology, C0121‐100 mL) for 20 min. The inserts were washed three times with phosphate‐buffered saline (PBS) and imaged under an inverted microscope. For the invasion assay, Matrigel (Corning, 356234) was diluted to 1 mg/mL in serum‐free medium, and 60 µL of the diluted Matrigel was added to the upper chamber and incubated at 37°C for 30 min. The subsequent procedures were the same as those used for the migration assay.

### 2.5 Western Blots

Western blotting was performed to assess protein expression. HCT116 and LoVo cells were lysed in radioimmunoprecipitation assay (RIPA) buffer (Beyotime Biotechnology, P0013B) supplemented with protease and phosphatase inhibitors (Beyotime Biotechnology, P1045). Protein concentrations were determined using a bicinchoninic acid (BCA) assay kit (Abcam, ab102536). Equal amounts of protein (30 µg) were resolved on 8%, 10%, or 4%–20% FuturePAGE precast gels (ACE Biotechnology, ET10008LGel, ET10010LGel, and ET10420LGel) according to protein size, and then transferred onto polyvinylidene difluoride (PVDF) membranes (Millipore, ISEQ00010). Membranes were blocked with 5% non‐fat milk in Tris‐buffered saline containing Tween 20 (TBST) for 1 h at room temperature, whereas 5% bovine serum albumin (BSA) in TBST was used for phosphorylated proteins. The membranes were incubated with primary antibodies overnight at 4°C, followed by horseradish peroxidase (HRP)‐conjugated secondary antibodies (1:10 000; Biodragon‐immunotech, BF03008 and BF03001) for 1 h at room temperature after TBST washing. Protein bands were detected using an enhanced chemiluminescence (ECL) detection kit (Biosharp, BL520A) and visualized with a chemiluminescence imaging system.

### 2.6 Co‐Immunoprecipitation

Co‐IP was performed to assess protein‐protein interactions. HCT116 and LoVo cells were lysed in IP lysis buffer (MedChemExpress, HY‐K1000) supplemented with protease and phosphatase inhibitors (MedChemExpress, HY‐K0010 and HY‐K0021). Protein concentration was measured using a BCA assay kit (Abcam, ab102536). For each reaction, 500 µg of total protein was incubated with 10 µL of the indicated primary antibody or the corresponding IgG control (Abcam, ab172730, ab18443, or ab18413) overnight at 4°C with gentle rotation. Pre‐washed Protein A/G magnetic beads (MedChemExpress, HY‐K0202) were then added and incubated for 2 h at 4°C. After five washes with ice‐cold IP lysis buffer, bound proteins were eluted with 2× SDS loading buffer at 95°C for 10 min, resolved by 10% sodium dodecyl sulfate–polyacrylamide gel electrophoresis (SDS‐PAGE), and subjected to western blotting. Signals were visualized using an ECL detection kit.

### 2.7 Denaturing Ubiquitination Assay

Cells were treated with MG132 (10 µM) for 6 h before harvesting. Cells were lysed in denaturing buffer containing 1% SDS, 10 mM N‐ethylmaleimide, and protease inhibitors and heated at 95°C for 10 min. After brief sonication, the lysates were diluted tenfold with IP buffer to reduce the final SDS concentration to 0.1% and subjected to immunoprecipitation with an anti‐Myc antibody or species‐matched control IgG. The immunoprecipitates were then analyzed by immunoblotting with an anti‐HA antibody to detect HA‐tagged ubiquitin conjugated to DDX3X.

### 2.8 Immunofluorescence

For immunofluorescence staining, 2000–4000 CRC cells were seeded onto glass coverslips and cultured overnight. Cells were fixed with 4% paraformaldehyde (Biosharp, BL539A) in PBS for 15 min at room temperature, followed by three washes with PBS. After permeabilization with 0.1% Triton X‐100 in PBS for 10 min at room temperature, cells were washed three more times with PBS. For R‐loop specificity validation, cells in the treatment group were incubated with RNase H (NEB, M0297S) at 37°C for 1–2 h, while the control group was incubated with the buffer alone. Then, cells were washed three more times with PBS and blocked with 5% BSA (MedChemExpress, HY‐D0842) in PBS for 1 h at room temperature to reduce nonspecific binding. Cells were then incubated with the indicated primary antibodies diluted 1:200 in 1% BSA at 4°C overnight. Following three washes with PBS, cells were incubated with the corresponding secondary antibodies diluted 1:500 in 1% BSA for 1 h at room temperature in the dark. Nuclei were counterstained with 4′,6‐diamidino‐2‐phenylindole (DAPI, Invitrogen, D1306) for 5 min. Coverslips were finally mounted with antifade mounting medium, and images were captured using a fluorescence microscope.

### 2.9 Immunohistochemical Staining

Formalin‐fixed, paraffin‐embedded (FFPE) CRC tissue sections (4 µm) were deparaffinized in xylene and rehydrated through a graded series of ethanol. After deparaffinization and rehydration, antigen retrieval was performed using citrate buffer (pH 6.0; Thermo Fisher Scientific, 005000). Endogenous peroxidase activity was quenched with 3% H_2_O_2_ for 10 min, followed by blocking with 5% BSA for 30 min at room temperature. The sections were incubated with the indicated primary antibodies overnight at 4°C. After washing three times with PBS, sections were incubated with HRP‐conjugated secondary antibodies for 30 min at room temperature. Signals were developed using the DAB+ Substrate Chromogen System (Agilent/Dako, K3468), and nuclei were counterstained with hematoxylin (Agilent/Dako, S3309). The stained sections were then dehydrated, mounted, and imaged under a light microscope.

### 2.10 Silver Staining of SDS‐PAGE Gels

After SDS‐PAGE, silver staining was performed using a Fast Silver Stain Kit (Thermo Scientific, 24612) according to the manufacturer's instructions. Briefly, the gel was sequentially fixed, sensitized, stained, and developed, with extensive washing in ultrapure water between each step. Development was closely monitored and terminated once distinct protein bands appeared. The gel was subsequently scanned for documentation.

### 2.11 DNA‐RNA Immunoprecipitation

Genomic DNA (gDNA) was extracted from HCT116 and LoVo cells using a commercial kit (TIANGEN, DP304) and treated with RNase T1 (Thermo Scientific, EN0541), ShortCut RNase III (New England Biolabs, M0245S), and RNase A (Thermo Scientific, EN0531) at 37°C for 1 h to remove free RNA species. The DNA was then sonicated to an average fragment size of approximately 200 bp. For immunoprecipitation, 7.5 µg of fragmented gDNA was incubated overnight at 4°C with 10 µL of S9.6 antibody or control antibody in binding buffer (10 mM sodium phosphate, 140 mM NaCl, and 0.05% Triton X‐100). Pre‐washed Protein A/G magnetic beads were added and incubated for 2 h at 4°C, followed by washing and elution in buffer containing 50 mM Tris‐HCl, 10 mM EDTA, and 1% SDS at 65°C for 15 min. For specificity controls, gDNA was treated with RNase H (New England Biolabs, M0297S; 1.7 U/µg DNA) at 37°C for 2 h before immunoprecipitation. Purified DNA was analyzed by quantitative polymerase chain reaction (qPCR) to determine R‐loop enrichment at the indicated genomic loci.

### 2.12 Electrophoretic Mobility Shift Assay (EMSA)

The R‐loop region was designed based on the DDX3X‐binding site within the GNG13 promoter. Two DNA oligonucleotides and one RNA oligonucleotide corresponding to the target sequence were synthesized and annealed at equimolar ratios in annealing buffer (90 mM Tris‐HCl, pH 7.5, 50 mM NaCl, and 10 mM MgCl2). After heating at 95°C for 5 min, the mixture was gradually cooled to room temperature at 0.1°C/s to generate R‐loop mimics. EMSA was performed using the LightShift Chemiluminescent EMSA Kit (Thermo Scientific, 20148) according to the manufacturer's instructions. The synthesized R‐loop mimics were incubated with purified DDX3X protein at 30°C for 15 min, separated on a 4% native polyacrylamide gel (YEASEN, 21102ES50), transferred to a nylon membrane, UV cross‐linked, and detected by chemiluminescence.

### 2.13 Sphere Formation Assay

HCT116 and LoVo cells were dissociated into single‐cell suspensions and cultured in serum‐free DMEM/F12 (Gibco, C11330500BT) supplemented with B‐27 (Gibco, 17504044), 20 ng/mL EGF (PeproTech, AF‐100‐15‐100), 20 ng/mL bFGF (PeproTech, 100–18B‐100ug), 5 µg/mL insulin (Gibco, 12585014), and 1% penicillin/streptomycin. Cells were seeded into ultra‐low attachment plates (Corning Costar, 3473) at the indicated density and maintained at 37°C with 5% CO_2_. Half of the medium was replenished every 3 days. After 7–10 days, spheres were imaged and quantified under a microscope, and only spheres >50 µm in diameter were included in the analysis. Sphere‐forming capacity was assessed by comparing sphere number and diameter among different groups.

### 2.14 S9.6 Dot Blot

Genomic DNA was extracted from CRC cells using a genomic DNA extraction kit and diluted in TE buffer. To minimize background and improve assay specificity, gDNA was pretreated with RNases A, T1, and III to remove contaminating single‐stranded and double‐stranded RNAs. The prepared DNA samples were then spotted onto a nylon membrane (Thermo Scientific, 77016) and UV cross‐linked. After blocking with 5% milk in TBST for 1 h at room temperature, the membranes were incubated overnight at 4°C with either S9.6 antibody (Kerafast, ENH001) or dsDNA antibody (Abcam, ab27156). Following TBST washes, the membranes were incubated with the corresponding HRP‐conjugated secondary antibody and subjected to chemiluminescent detection.

### 2.15 Expression and Purification of the ND‐iRGD Fusion Peptide

The sequence encoding NDFIP2 amino acids 147–220 was fused in‐frame with iRGD and cloned into the His‐tagged pET‐28a vector, following previously described strategies [14]. After sequence verification, the construct was expressed in E. coli BL21 (DE3) by induction with 0.2 × 10^−3^ M IPTG at 16°C for 16 h. The soluble fusion peptide was purified by Ni^2+^‐NTA affinity and size‐exclusion chromatography, followed by endotoxin removal and buffer exchange into sterile PBS. Purity was confirmed by SDS‐PAGE, and the purified peptide was stored at −80°C until use.

### 2.16 Tail Vein Injection Metastasis Assay

HCT116‐luc cells were harvested, washed twice with PBS, and resuspended in sterile PBS. BALB/c nude mice aged 4–6 weeks were intravenously injected via the lateral tail vein with 1 × 10^6^ cells suspended in 100 µL PBS per mouse. Beginning 1 week after tumor cell inoculation, mice received intraperitoneal injections of iRGD or the ND‐iRGD fusion peptide at 25 µmol/kg every 3 days for 2 weeks. Three weeks after injection, lung metastasis was assessed by in vivo bioluminescence imaging. Prior to imaging, mice were anesthetized with isoflurane and intraperitoneally administered D‐luciferin at 150 mg/kg (MedChemExpress, HY‐12591B). Bioluminescent signals were captured using an IVIS imaging system.

### 2.17 Splenic Injection‐Induced Liver Metastasis Assay

HCT116‐luc cells were harvested, washed twice with PBS, and resuspended in sterile PBS. BALB/c nude mice aged 4–6 weeks were anesthetized with isoflurane, and a small subcostal incision was made on the left flank to expose the spleen. Approximately 1 × 10^6^ cells suspended in 50 µL PBS were slowly injected into the splenic pulp. To prevent the formation of primary splenic tumors and ensure the portal dissemination of cancer cells, the splenic pedicle was ligated, and a total splenectomy was performed 5 min after the injection. The abdominal wall and skin were then sutured. Beginning 1 week after tumor cell inoculation, mice received intraperitoneal injections of iRGD or ND‐iRGD at 25 µmol/kg every 3 days for 2 weeks. Three weeks after tumor cell inoculation, liver metastasis was assessed by in vivo bioluminescence imaging. Mice were intraperitoneally administered D‐luciferin (150 mg/kg; MedChemExpress, HY‐12591B), and bioluminescent signals were captured using an IVIS imaging system to quantify the metastatic burden in the liver. All animal experiments were reviewed and approved by the Laboratory Animal Ethics Committee of Xuzhou Medical University (approval number: 202503T034) and were performed in accordance with the institutional guidelines for the care and use of laboratory animals.

### 2.18 Statistical Analysis

Statistical analyses were performed using GraphPad Prism 10.0 (RRID: SCR_002798). Data were analyzed without transformation or normalization unless otherwise specified, and no data points were excluded as outliers. Data are presented as the mean ± SD or median with interquartile range (IQR), as indicated in the corresponding figure legends. Comparisons between two groups were performed using two‐tailed unpaired Student's t‐test, Welch's t‐test, or Mann–Whitney U test, as appropriate. Comparisons among multiple groups were performed using one‐way or two‐way ANOVA followed by Tukey's, Dunnett's, or Šídák's multiple‐comparisons test, as appropriate and as specified in the corresponding figure legends. Survival differences were evaluated using the log‐rank (Mantel–Cox) test. In vitro experiments were independently repeated at least three times unless otherwise specified, and the exact sample size (n) and statistical test used for each analysis are provided in the corresponding figure legends. All statistical tests were two‐sided unless otherwise specified. A *p* value < 0.05 was considered statistically significant; ns, not significant; ^*^ *p* < 0.05, ^**^ *p* < 0.01, ^***^ *p* < 0.001, ^****^ *p* < 0.0001.

## 3 Results

### 3.1 Low NDFIP2 Expression is Closely Associated With CRC Metastasis and a Poor Prognosis

To investigate the molecular mechanisms underlying CRC metastasis, we analyzed primary tumors from 12 patients, including those with (T2N0M1) and without (T2N0M0) distant metastasis. Bulk RNA‐seq was performed on six samples, including three T2N0M1 and three T2N0M0 cases, identifying 1076 genes significantly downregulated in metastatic tumors (Figure 1A). In parallel, single‐cell RNA‐seq was conducted on an independent cohort of six tumors including three T2N0M1 and three T2N0M0 cases, revealing 642 downregulated and 544 upregulated genes in the metastatic group (Figure 1B). Integrative analysis of bulk and single‐cell RNA‐seq data identified 9 genes negatively and 5 genes positively associated with CRC metastasis. Further intersection with metastasis‐related genes from the CancerSEA database [15] identified NDFIP2 as the sole overlapping candidate (Figure 1C). Consistently, both mRNA and protein analyses confirmed significantly reduced NDFIP2 expression in T2N0M1 tumors compared with T2N0M0 tumors (Figure S1A,B). CancerSEA analysis further demonstrated a negative correlation between NDFIP2 expression and CRC metastasis (Figure 1D), suggesting a potential metastasis‐suppressive role. Single‐cell analysis revealed that NDFIP2 is broadly expressed across multiple cell types, with the highest levels observed in tumor epithelial cells, followed by cancer‐associated fibroblasts (CAFs) and T cells (Figure 1E,F).

**FIGURE 1:**
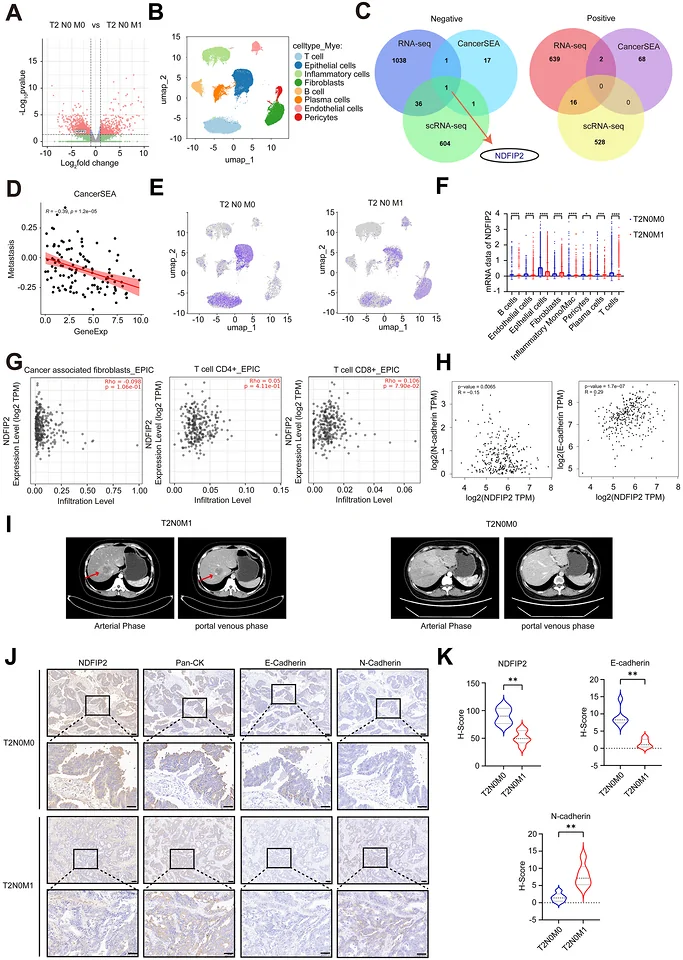
Low NDFIP2 expression is closely associated with CRC metastasis and a poor prognosis. (A) Volcano plot showing differentially expressed genes between T2N0M0 and T2N0M1 colorectal cancer tissues based on bulk RNA‐seq analysis. Red dots represent significantly differentially expressed genes. (B) UMAP plot of scRNA‐seq data showing the major cell populations in CRC tissues, including T cells, epithelial cells, inflammatory cells, fibroblasts, B cells, plasma cells, endothelial cells, and pericytes. (C) Venn diagrams showing the overlap of candidate genes negatively or positively associated with metastasis identified from bulk RNA‐seq, scRNA‐seq, and CancerSEA analyses. (D) Correlation between NDFIP2 expression and CRC metastasis based on the CancerSEA database. (E) UMAP feature plots showing NDFIP2 expression across different cell types in T2N0M0 and T2N0M1 CRC tissues. (F) Cell type‐specific analysis of NDFIP2 mRNA expression across different cellular populations identified by scRNA‐seq from T2N0M0 (n = 3 patients) and T2N0M1 (n = 3 patients) CRC tissues. Data are presented as median with interquartile range (IQR). Statistical significance was determined using a two‐sided Wilcoxon rank‐sum test. ^*^ *p* < 0.05, ^***^ *p* < 0.001, and ^****^ *p* < 0.0001. (G) Correlation analyses between NDFIP2 expression and infiltration levels of cancer‐associated fibroblasts, CD4^+^ T cells, and CD8^+^ T cells, as estimated by the EPIC algorithm. (H) Dot plots showing the correlations of NDFIP2 expression with E‐cadherin and N‐cadherin mRNA expression in the TCGA CRC dataset (GEPIA2). (I) Representative contrast‐enhanced CT images in the arterial phase and portal venous phase from patients with T2N0M1 and T2N0M0 CRC. Red arrows indicate metastatic lesions. (J‐K) Representative immunohistochemical staining (J) and corresponding H‐score quantification (K) of NDFIP2, E‐cadherin, and N‐cadherin in serial sections of primary CRC tissues from patients with T2N0M0 or T2N0M1 disease. The boxed regions are shown at higher magnification in the corresponding lower panels. Scale bars: 100 µm for the original images and 50 µm for the enlarged images. Pan‐CK staining was used to delineate epithelial tumor regions, and the H‐scores of NDFIP2, E‐cadherin, and N‐cadherin were calculated exclusively within the spatially matched Pan‐CK‐positive regions. Data are presented as median with interquartile range (n = 6). Statistical significance was determined using a two‐tailed Mann‐Whitney U test. ^**^ *p* < 0.01.

EPIC analysis showed no significant correlation between NDFIP2 expression and the infiltration of CAFs or T cells (Figure 1G), suggesting that NDFIP2 may primarily regulate CRC metastasis in epithelial cells. Consistently, GEPIA2 analysis revealed that NDFIP2 expression positively correlated with E‐cadherin and negatively with N‐cadherin [16], indicating a potential link to EMT status (Figure 1H).

CT imaging showed an evident metastatic lesion in the patient with T2N0M1, whereas no distant metastasis was detected in the patient with T2N0M0 (Figure 1I). Immunohistochemical analyses further confirmed that NDFIP2 was predominantly expressed in epithelial tumor cells. Compared with T2N0M0 tumors, NDFIP2 expression was significantly reduced in the CRC epithelial cells of T2N0M1 tumors, whereas no apparent difference was observed in the surrounding non‐epithelial cells. T2N0M1 tumors also exhibited decreased E‐cadherin expression and increased N‐cadherin expression (Figure 1J,K). Collectively, these findings suggest that NDFIP2 is closely associated with the metastatic potential of CRC epithelial cells.

We next evaluated the prognostic significance of NDFIP2 in CRC. Kaplan‐Meier Plotter analysis [17] revealed that high NDFIP2 expression was significantly associated with improved relapse‐free survival, but not overall survival (Figure S1C).

### 3.2 NDFIP2 Suppresses Colorectal Cancer Metastasis by Inhibiting PI3K/AKT Signaling

To investigate the role of NDFIP2 in CRC metastasis, we generated NDFIP2‐knockout HCT116 and LoVo cells using CRISPR‐Cas9 [18], with efficient depletion confirmed by western blotting (Figure 2A and Figure S2A). Transwell assays showed that NDFIP2 knockout significantly enhanced cell migration and invasion, whereas NDFIP2 overexpression exerted the opposite effects (Figure 2B,C and Figure S2B–D). Consistently, NDFIP2 depletion promoted epithelial‐mesenchymal transition (EMT), as evidenced by increased N‐cadherin and decreased E‐cadherin expression, while NDFIP2 overexpression reversed these changes (Figure 2D,E).

**FIGURE 2:**
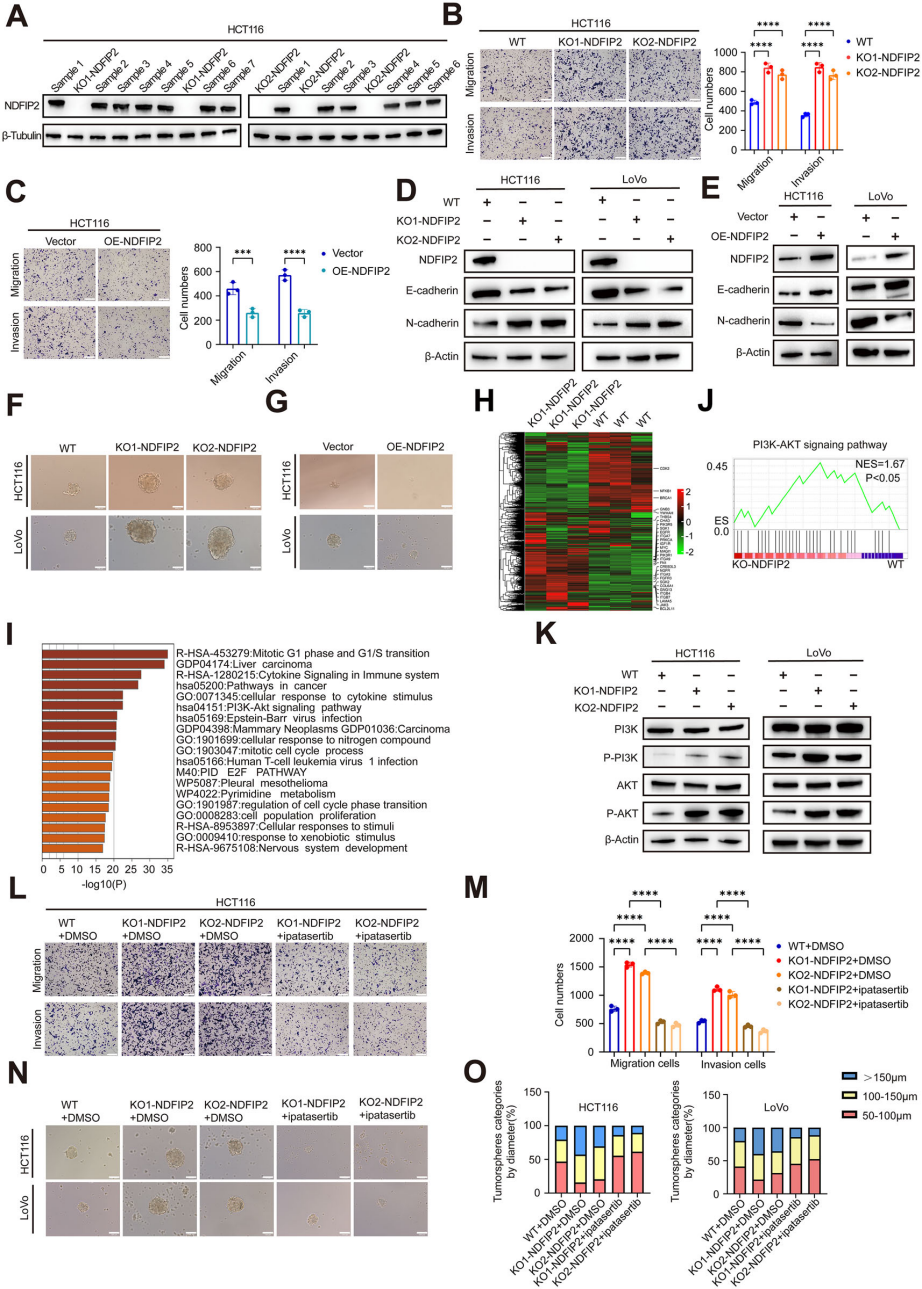
NDFIP2 suppresses CRC migration, invasion, and stemness by inhibiting PI3K/AKT signaling. (A) Immunoblot validation of NDFIP2 knockout efficiency in independently generated HCT116 cell clones. Tubulin served as a loading control. (B) Representative images and quantification of Transwell migration and invasion assays in HCT116 cells with wild‐type control (WT) or NDFIP2 knockout (KO1‐NDFIP2 and KO2‐NDFIP2). Scale bar: 200 µm. Data are presented as mean ± SD (n = 3). Statistical significance was determined using one‐way ANOVA followed by Tukey's multiple comparisons test. ^****^ *p* < 0.0001. (C) Representative images and quantification of Transwell migration and invasion assays in HCT116 cells transfected with empty vector or NDFIP2 overexpression construct (OE‐NDFIP2). Scale bar: 200 µm. Data are presented as mean ± SD (n = 3). Statistical significance was determined using a two‐tailed unpaired Student's t‐test. ^***^ *p* < 0.001,^****^ *p* < 0.0001. (D) Immunoblot analysis of NDFIP2, E‐cadherin, and N‐cadherin in HCT116 and LoVo cells with WT, KO1‐NDFIP2, or KO2‐NDFIP2. β‐Actin served as a loading control. (E) Immunoblot analysis of NDFIP2, E‐cadherin, and N‐cadherin in HCT116 and LoVo cells with Vector or OE‐NDFIP2. β‐Actin served as a loading control. (F) Representative images of tumorsphere formation in HCT116 and LoVo cells with WT, KO1‐NDFIP2, or KO2‐NDFIP2. Scale bar: 100 µm. (G) Representative images of tumorsphere formation in HCT116 and LoVo cells transfected with empty vector or OE‐NDFIP2. Scale bar: 100 µm. (H) Heatmap showing differentially expressed genes between KO1‐NDFIP2 and WT HCT116 cells based on RNA‐sequencing analysis. (I) Pathway enrichment analysis of differentially expressed genes identified by RNA sequencing. (J) GSEA showing that the genes that were differentially expressed after NDFIP2 knockout were enriched in the PI3K/AKT signaling gene set. (K) Immunoblot analysis of PI3K, phospho‐PI3K (p‐PI3K), AKT, and phospho‐AKT (p‐AKT) in HCT116 and LoVo cells with NDFIP2 knockout. β‐Actin served as a loading control. (L,M) Representative images (L) and quantification (M) of Transwell migration and invasion assays in HCT116 cells treated with DMSO or the AKT inhibitor ipatasertib after NDFIP2 knockout. Scale bar: 200 µm. Data are presented as mean ± SD (n = 3 independent experiments). Statistical significance was determined using one‐way ANOVA followed by Tukey's multiple comparisons test. ^****^ *p* < 0.0001. (N) Representative images of tumorsphere formation in HCT116 and LoVo cells treated with DMSO or ipatasertib after NDFIP2 knockout. Scale bar: 100 µm. (O) Quantification of tumorsphere diameter distribution in HCT116 and LoVo cells under the indicated treatments.

Given the role of cancer stemness in metastasis [19], we next assessed whether NDFIP2 regulates stem‐like properties. Tumorsphere assays [20] revealed that NDFIP2 knockout markedly increased sphere formation, whereas NDFIP2 overexpression suppressed both sphere number and size [21] (Figure 2F,G and Figure S2E–H). Consistently, NDFIP2 depletion upregulated stemness‐associated markers, including CD133, CD44, and OCT4, while NDFIP2 overexpression reduced their expression (Figure S3A–D).

To elucidate the underlying mechanisms, RNA‐seq was performed in NDFIP2‐knockout and control CRC cells (Figure 2H). The PI3K/AKT signaling pathway is a well‐established regulatory axis closely associated with tumor metastasis. Although our KEGG pathway analysis did not identify it as the most significantly enriched pathway, it ranked among the top enriched pathways. In contrast, the other highly ranked terms, such as cytokine‐related pathways, are primarily associated with the tumor microenvironment, which falls beyond the scope of our cell‐based experimental system. We therefore selected the PI3K/AKT signaling pathway as the focus of the present study (Figure 2I,J). Accordingly, NDFIP2 knockout increased the levels of phosphorylated PI3K and AKT, whereas NDFIP2 overexpression suppressed their activation [22] (Figure 2K and Figure S3E,F), indicating that NDFIP2 negatively regulates PI3K/AKT signaling.

To determine whether PI3K/AKT activation mediates the pro‐metastatic effects of NDFIP2 loss, cells were treated with the AKT inhibitor ipatasertib [23]. We evaluated the effects of ipatasertib on the malignant phenotypes of CRC cells, consistent with its previously reported antitumor activity in colon cancer [24]. In vitro, Cell Counting Kit‐8 (CCK‐8) and Transwell assays demonstrated that ipatasertib significantly suppressed CRC cell proliferation, migration, and invasion (Figure S3G–I). Consistently, in vivo metastasis assays further demonstrated that ipatasertib markedly impaired the metastatic capacity of CRC cells (Figure S3J,K). Importantly, pharmacological inhibition of AKT largely reversed NDFIP2 knockout‐induced increases in migration, invasion, and sphere formation (Figure 2L–O and Figure S3L–O). Consistently, ipatasertib abolished the NDFIP2 knockout‐induced upregulation of N‐cadherin, stemness‐associated markers and p‐PRAS40, while reversing the concomitant downregulation of E‐cadherin (Figure S3P).

We next evaluated the role of NDFIP2 in vivo using a lung metastasis model [25]. NDFIP2 knockout significantly increased metastatic burden, as indicated by higher incidence, greater numbers of lung nodules, and enhanced fluorescence signal intensity (Figure 3A–C). Notably, ipatasertib treatment markedly attenuated these effects. Immunohistochemical analysis revealed that NDFIP2 knockout downregulated E‐cadherin and upregulated N‐cadherin, p‐PRAS40, and CD133 expression. Notably, these alterations were effectively reversed by ipatasertib treatment (Figure 3D and Figure S4A). Moreover, NDFIP2 knockout shortened overall survival compared to the WT group, whereas ipatasertib treatment rescued the survival disadvantage caused by NDFIP2 loss (Figure 3E). Accordingly, we evaluated the role of NDFIP2 in vivo by establishing a nude mouse liver metastasis model. In vivo fluorescence imaging confirmed that NDFIP2 knockout significantly enhanced liver metastasis, an effect that was markedly inhibited by ipatasertib treatment (Figure 3F). Furthermore, the number of liver metastatic nodules was significantly increased following NDFIP2 knockout, and this increase was similarly reversed by ipatasertib (Figure 3G,H). Next, we performed immunohistochemical analysis on the liver metastatic lesions from each group. The results confirmed that NDFIP2 knockout led to a significant upregulation of p‐PRAS40, N‐cadherin, and CD133 expression. However, these levels notably declined in the KO‐NDFIP2 + ipatasertib group. Conversely, E‐cadherin expression was significantly downregulated in the KO‐NDFIP2 group, while its expression levels effectively recovered following combined treatment with ipatasertib (Figure 3I and Figure S4B). Survival analysis demonstrated that NDFIP2 knockout significantly shortened the overall survival of the mice, whereas ipatasertib treatment effectively suppressed this effect (Figure 3J).

**FIGURE 3:**
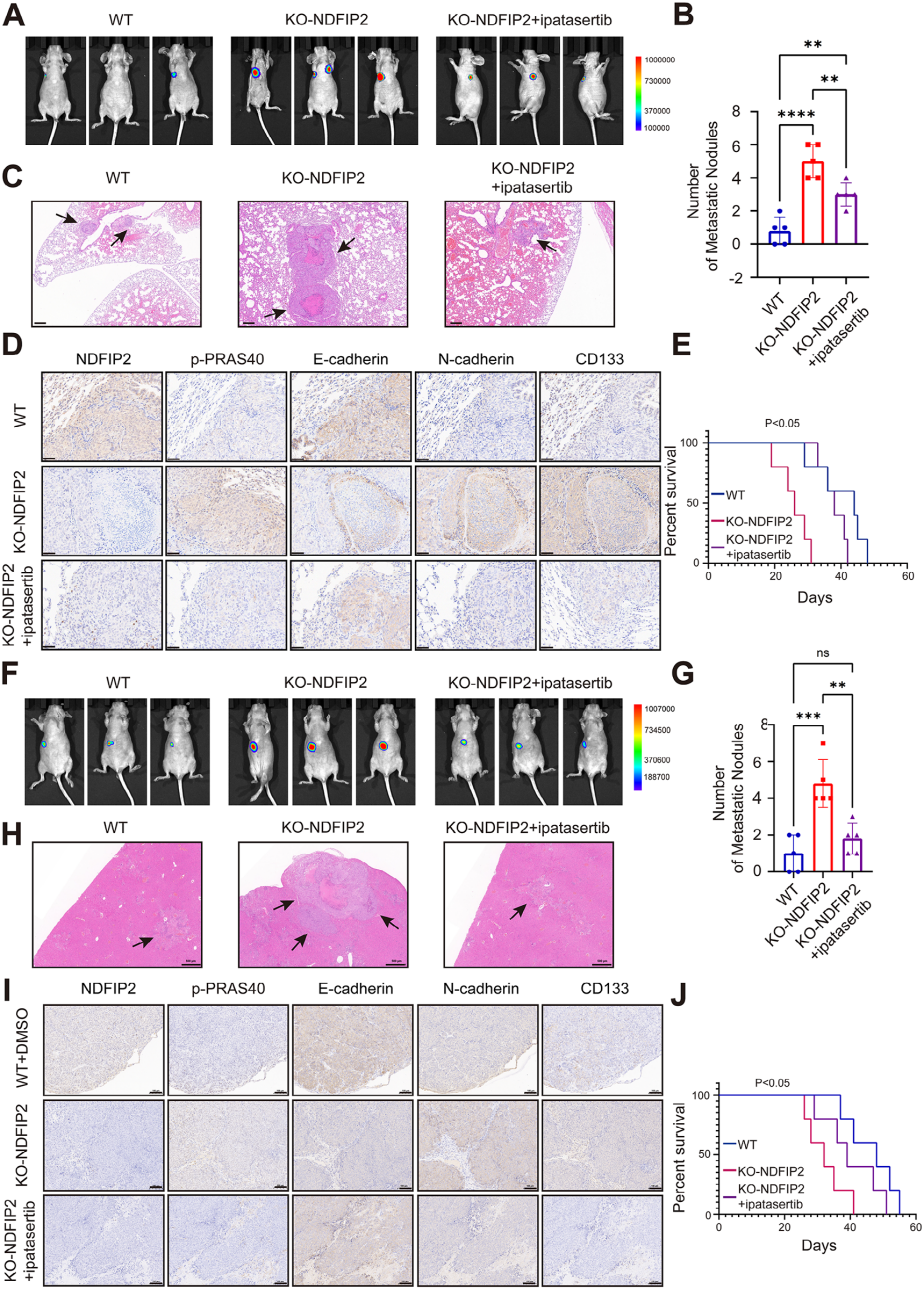
NDFIP2 suppresses CRC metastasis in vivo through PI3K/AKT signaling. (A) Representative bioluminescent images of lung metastasis acquired through IVIS three weeks after injection from the WT (n  =  5), KO‐NDFIP2 (n  =  5), and KO‐NDFIP2 + ipatasertib groups; (B) Quantification of the number of metastatic lung nodules in each group. Data are presented as mean ± SD (n = 5). Statistical significance was determined using one‐way ANOVA followed by Tukey's multiple comparisons test. ^**^ *p* < 0.01, ^****^ *p* < 0.0001. (C) Representative hematoxylin and eosin (H&E) staining of lung sections from mice in each group. Arrows indicate metastatic lesions. Scale bar: 200 µm. (D) Representative immunohistochemical staining of NDFIP2, p‐PRAS40, E‐cadherin, N‐cadherin, and CD133 in lung metastatic lesions from the indicated groups. Scale bar: 50 µm. (E) Kaplan‒Meier analysis showing the survival period of the mice in each group (n  =  5). Statistical significance was determined using the log‐rank (Mantel–Cox) test. P < 0.05 was considered statistically significant. (F) Representative bioluminescent images of liver metastasis acquired through IVIS three weeks after injection from the WT (n  =  5), KO‐NDFIP2 (n  =  5), and KO‐NDFIP2 + ipatasertib groups; (G) Quantification of the number of metastatic liver nodules in each group. Data are presented as mean ± SD (n = 5). Statistical significance was determined using one‐way ANOVA followed by Tukey's multiple comparisons test. ns, not significant; ^**^ *p* < 0.01, ^***^ *p* < 0.001, ^****^ *p* < 0.0001. (H) Representative hematoxylin and eosin (H&E) staining of liver sections from mice in each group. Arrows indicate metastatic lesions. Scale bar: 500 µm. (I) Representative immunohistochemical staining of NDFIP2, p‐PRAS40, E‐cadherin, N‐cadherin, and CD133 in liver metastatic lesions from the indicated groups. Scale bar: 100 µm. (J) Kaplan‒Meier analysis showing the survival period of the mice in each group (n  =  5). Statistical significance was determined using the log‐rank (Mantel–Cox) test. P < 0.05 was considered statistically significant.

Collectively, these results demonstrate that NDFIP2 suppresses CRC metastasis by inhibiting PI3K/AKT signaling in vitro and in vivo.

### 3.3 NDFIP2 Interacts With DDX3X and Promotes its K48‐linked Polyubiquitination and Proteasomal Degradation, Thereby Suppressing PI3K/AKT Signaling

To elucidate how NDFIP2 suppresses PI3K/AKT signaling, we performed immunoprecipitation (IP) followed by silver staining to identify NDFIP2‐interacting proteins (Figure 4A). Mass spectrometry analysis identified 412 candidate interactors (Table S1), among which DDX3X ranked prominently after excluding cytoskeletal contaminants. Co‐immunoprecipitation assays confirmed the interaction between NDFIP2 and DDX3X (Figure S5A,B), which was further supported by their partial colocalization in immunofluorescence analyses (Figure 4B). Functionally, DDX3X knockdown largely reversed the activation of PI3K/AKT signaling induced by NDFIP2 depletion (Figure 4C), indicating that NDFIP2 regulates this pathway in a DDX3X‐dependent manner. Consistent with this finding, DDX3X knockdown substantially reversed the alterations in markers associated with EMT and stemness caused by NDFIP2 depletion, attenuating the upregulation of N‐cadherin, CD133, and OCT4 while restoring E‐cadherin expression (Figure S5C). Moreover, migration and invasion assays showed that DDX3X knockdown markedly attenuated the enhanced migratory and invasive capacities of NDFIP2‐deficient CRC cells (Figure S5D,E). Similarly, tumorsphere formation assays demonstrated that DDX3X depletion significantly reversed the increased sphere‐forming capacity induced by NDFIP2 loss (Figure S5F–H). Collectively, these findings establish DDX3X as a critical downstream mediator of the pro‐metastatic and stemness‐promoting phenotypes resulting from NDFIP2 depletion. Moreover, we performed immunohistochemical analysis and demonstrated that DDX3X, p‐AKT, and CD133 expression was significantly elevated in T2N0M1 tumors compared with T2N0M0 tumors, further substantiating our findings (Figure S5I,J). We next sought to elucidate the molecular mechanism by which NDFIP2 regulates DDX3X. Western blotting and qPCR analyses demonstrated that NDFIP2 negatively regulated DDX3X at the protein level, without altering its mRNA expression (Figure 4D,E and Figure S5K). Further studies demonstrated that NDFIP2 knockout prolonged the half‐life of DDX3X, whereas NDFIP2 overexpression markedly accelerated its degradation [26] (Figure 4F–H). To determine the degradation pathway involved, cells were treated with the proteasome inhibitor MG132 [27] or the lysosomal inhibitor chloroquine. Follow‐up experiments revealed that MG132, but not chloroquine, blocked NDFIP2‐induced DDX3X degradation, suggesting that NDFIP2 mediates DDX3X degradation through a proteasome‐dependent pathway [28] (Figure 4I). Ubiquitination assays showed that NDFIP2 overexpression markedly enhanced DDX3X ubiquitination, whereas NDFIP2 depletion reduced it (Figure 4J).

**FIGURE 4:**
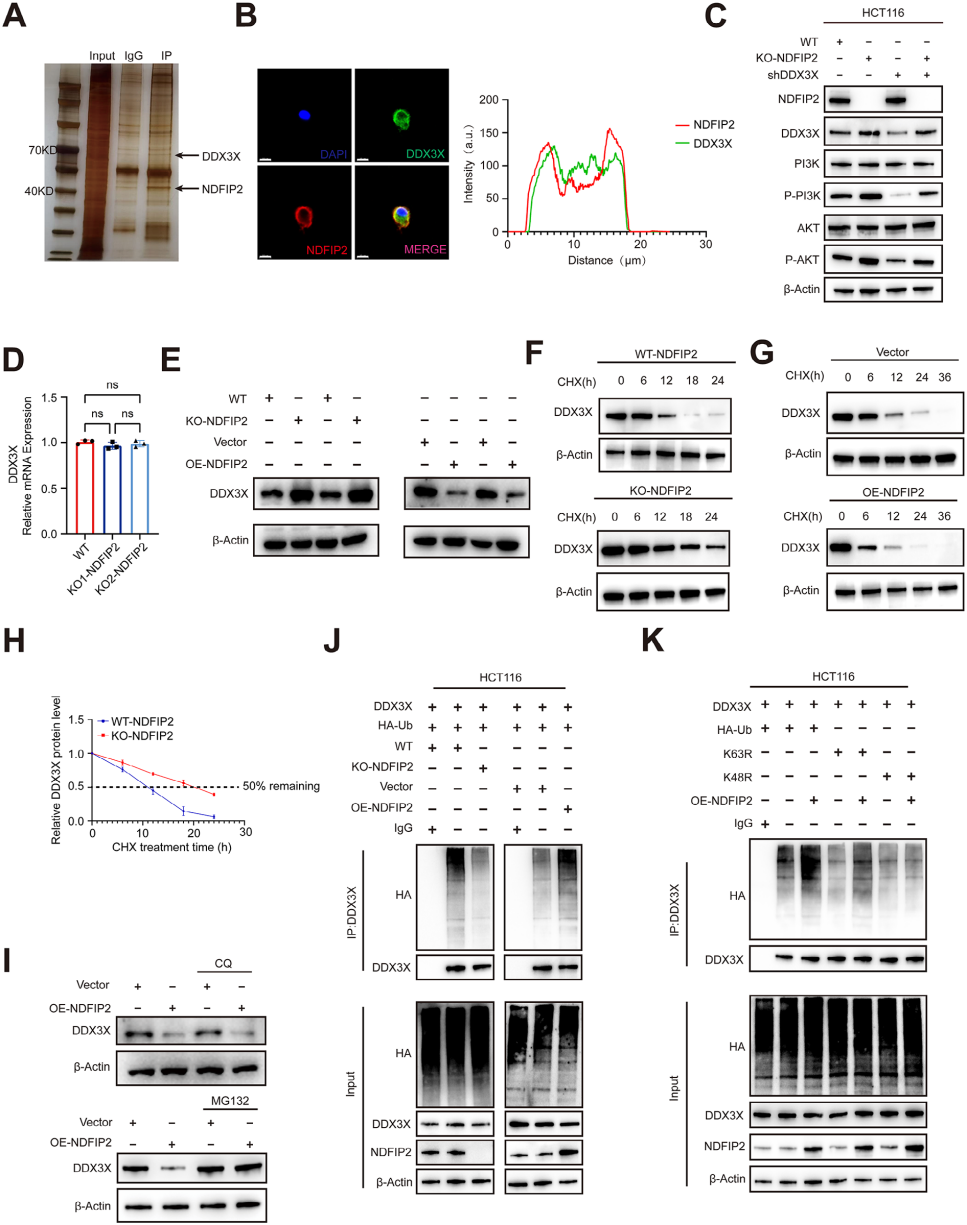
NDFIP2 interacts with DDX3X and promotes its K48‐linked polyubiquitination and proteasomal degradation, thereby suppressing PI3K/AKT signaling. (A) Silver staining of proteins in input, IgG, and IP fractions, showing the bands corresponding to DDX3X and NDFIP2. (B) Immunofluorescence staining and line‐scan analysis showing the partial colocalization of NDFIP2 and DDX3X. Scale bar: 10 µm. (C) Immunoblot analysis showing that DDX3X knockdown attenuated PI3K/AKT activation induced by NDFIP2 loss in HCT116 cells. WT and NDFIP2‐KO HCT116 cells were transduced with either shNC or shDDX3X, as indicated. (D) qRT‐PCR analysis of DDX3X mRNA expression in WT and NDFIP2‐knockout cells. Data are presented as mean ± SD (n = 3). Statistical significance was determined using one‐way ANOVA followed by Tukey's multiple comparisons test. ns, not significant. (E) Immunoblot analysis of DDX3X protein levels in NDFIP2‐knockout or NDFIP2‐overexpressing cells. (F,G) CHX chase assays showing the effects of NDFIP2 depletion or overexpression on DDX3X protein stability. (H) Densitometric quantification of the cycloheximide chase assays shown in (F). DDX3X protein levels were normalized to β‐actin and subsequently normalized to the corresponding level at 0 h. The half‐life of DDX3X was determined as the time required for its relative protein level to decrease to 50%, as indicated by the dashed line. The estimated DDX3X half‐life increased from 8.91 h in the WT‐NDFIP2 group to 20.74 h in the KO‐NDFIP2 group. (I) Immunoblot analysis showing that NDFIP2‐mediated DDX3X downregulation was blocked by treatment with MG132 (10 µM, 6 h), but not by CQ (10 µM, 12 h). (J) Ubiquitination assay showing that NDFIP2 promotes DDX3X polyubiquitination. (K) Ubiquitination assay with the indicated ubiquitin mutants showing that NDFIP2 promotes K48‐linked ubiquitination of DDX3X. All experiments shown in panels D‐K were performed using HCT116 cells.

To determine the type of ubiquitin linkage involved in this process [29], we employed ubiquitin mutants with lysine‐to‐arginine substitutions at K48 or K63. NDFIP2‐induced ubiquitination of DDX3X was preserved in the presence of wild‐type or K63R ubiquitin, but abolished by the K48R mutant (Figure 4K), indicating that NDFIP2 promotes K48‐linked polyubiquitination.

Collectively, these results demonstrate that NDFIP2 facilitates K48‐linked ubiquitination and proteasomal degradation of DDX3X, thereby suppressing PI3K/AKT signaling.

### 3.4 NDFIP2 Recruits NEDD4 via its PY Motifs to Promote DDX3X Ubiquitination

To further elucidate how NDFIP2 promotes DDX3X ubiquitination, we queried the UbiBrowser database to predict candidate E3 ligases targeting DDX3X. NEDD4 [30] ranked among the top candidates (Figure 5A). Given that previous studies have shown that NDFIP2 exerts its functions through interaction with NEDD4, we speculated that NEDD4 might be the key E3 ubiquitin ligase involved in this process. Co‐immunoprecipitation assays confirmed that NDFIP2 interacts with both DDX3X and NEDD4 (Figure 5B–D and Figure S6A). Their partial colocalization was further confirmed by immunofluorescence analyses (Figure 5E and Figure S6B,C).

**FIGURE 5:**
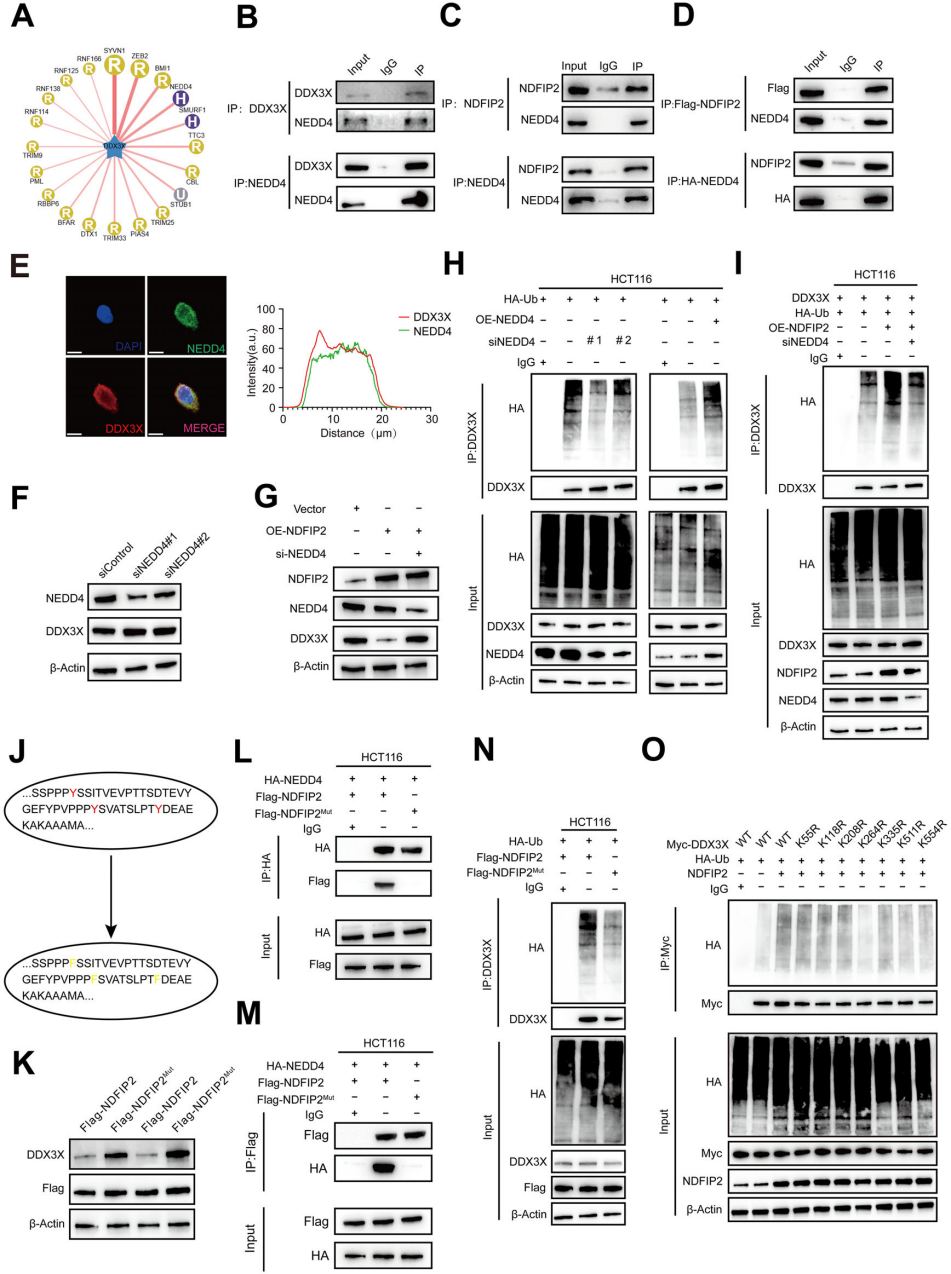
NDFIP2 recruits NEDD4 via its PY motifs to promote DDX3X ubiquitination. (A) Predicted E3 ligases potentially involved in DDX3X ubiquitination. (B) Endogenous co‐IP showing the interaction between DDX3X and NEDD4. (C,D) Endogenous and exogenous co‐IP showing the interaction between NDFIP2 and NEDD4. (E) Immunofluorescence staining and line‐scan analysis showing the co‐localization of DDX3X and NEDD4. Scale bar: 10 µm. (F) Immunoblot analysis of DDX3X expression after NEDD4 knockdown. (G) Immunoblot analysis showing that NEDD4 knockdown attenuated NDFIP2‐induced DDX3X downregulation. (H) Ubiquitination assays showing that NEDD4 depletion reduced, whereas NEDD4 overexpression increased, DDX3X ubiquitination. (I) Ubiquitination assay showing that NEDD4 knockdown attenuated NDFIP2‐enhanced DDX3X ubiquitination. (J) Schematic of the NDFIP2 PY‐motif mutant. (K) Immunoblot analysis showing that PY‐motif mutation impaired the ability of NDFIP2 to reduce DDX3X protein levels. (L,M) Co‐IP assays showing that PY‐motif mutation impaired the interaction between NDFIP2 and NEDD4. (N) Ubiquitination assay showing that the PY‐motif mutant attenuated NDFIP2‐induced DDX3X ubiquitination. (O) Ubiquitination assays with the indicated DDX3X lysine mutants identifying K264 as a major site required for NDFIP2‐mediated ubiquitination. All experiments shown in panels B‐O were performed using HCT116 cells.

Furthermore, NEDD4 knockdown increased DDX3X protein levels and reduced its ubiquitination, whereas NEDD4 overexpression exerted the opposite effects. NEDD4 silencing largely rescued the NDFIP2 overexpression‐induced decrease in DDX3X protein abundance and increase in its ubiquitination (Figure 5F–I and Figure S6D), indicating that NDFIP2‐mediated ubiquitination of DDX3X is dependent on NEDD4.

Previous studies have shown that the interaction between NDFIP2 and NEDD4 is mediated by the PY motifs of NDFIP2 [31]. To validate this interaction, we generated an NDFIP2 mutant in which all three PY motifs were disrupted by substituting tyrosine residues with phenylalanine (Figure 5J). Compared with wild‐type NDFIP2, the PY‐mutant failed to reduce DDX3X protein levels and showed markedly diminished interaction with NEDD4 (Figure 5K–M). Notably, disruption of the PY motifs also impaired the association between DDX3X and NEDD4 (Figure S6E) and significantly reduced DDX3X ubiquitination (Figure 5N), demonstrating that NDFIP2 recruits NEDD4 via its PY motifs to facilitate DDX3X ubiquitination.

To identify the specific ubiquitination site, we screened candidate lysine residues of DDX3X based on UbiBrowser predictions and generated a panel of lysine‐to‐arginine mutants (K55R, K118R, K208R, K264R, K335R, K551R, and K554R). NDFIP2 overexpression enhanced ubiquitination of all mutants except K264R (Figure 5O), indicating that K264 is the primary ubiquitination site. These results indicate that NDFIP2 regulates DDX3X ubiquitination through the K264 site of DDX3X.

Collectively, these findings indicate that NDFIP2 recruits NEDD4 to promote K48‐linked ubiquitination of DDX3X at K264, thereby accelerating its proteasomal degradation.

### 3.5 DDX3X Resolves Promoter‐Associated R‐Loops to Enhance GNG13 Transcription

DDX3X, a prominent member of the DEAD‐box RNA helicase family [32], operates as an ATP‐dependent enzyme orchestrating diverse aspects of RNA metabolism. To delineate the molecular basis underlying the functional interplay between DDX3X and PI3K/AKT signaling, we performed RNA‐seq analysis in HCT116 cells and identified a set of differentially expressed genes (DEGs). DEGs were defined as genes with |log_2_ fold change| ≥ 2 and an adjusted *p* value < 0.05. Genes were annotated to the PI3K/AKT signaling pathway using the KEGG gene set hsa04151, and the top five DEGs within this pathway were ranked by the magnitude of |log_2_ fold change|. Among the five candidates, GNG13 was the only gene with documented evidence of directly participating in PI3K activation [33], whereas the other four genes have primarily been reported as downstream effectors of PI3K signaling. GNG13 was therefore selected for further mechanistic investigation (Figure 6A), prompting us to investigate whether GNG13 acts as a downstream transcriptional effector through which DDX3X drives CRC metastasis.

**FIGURE 6:**
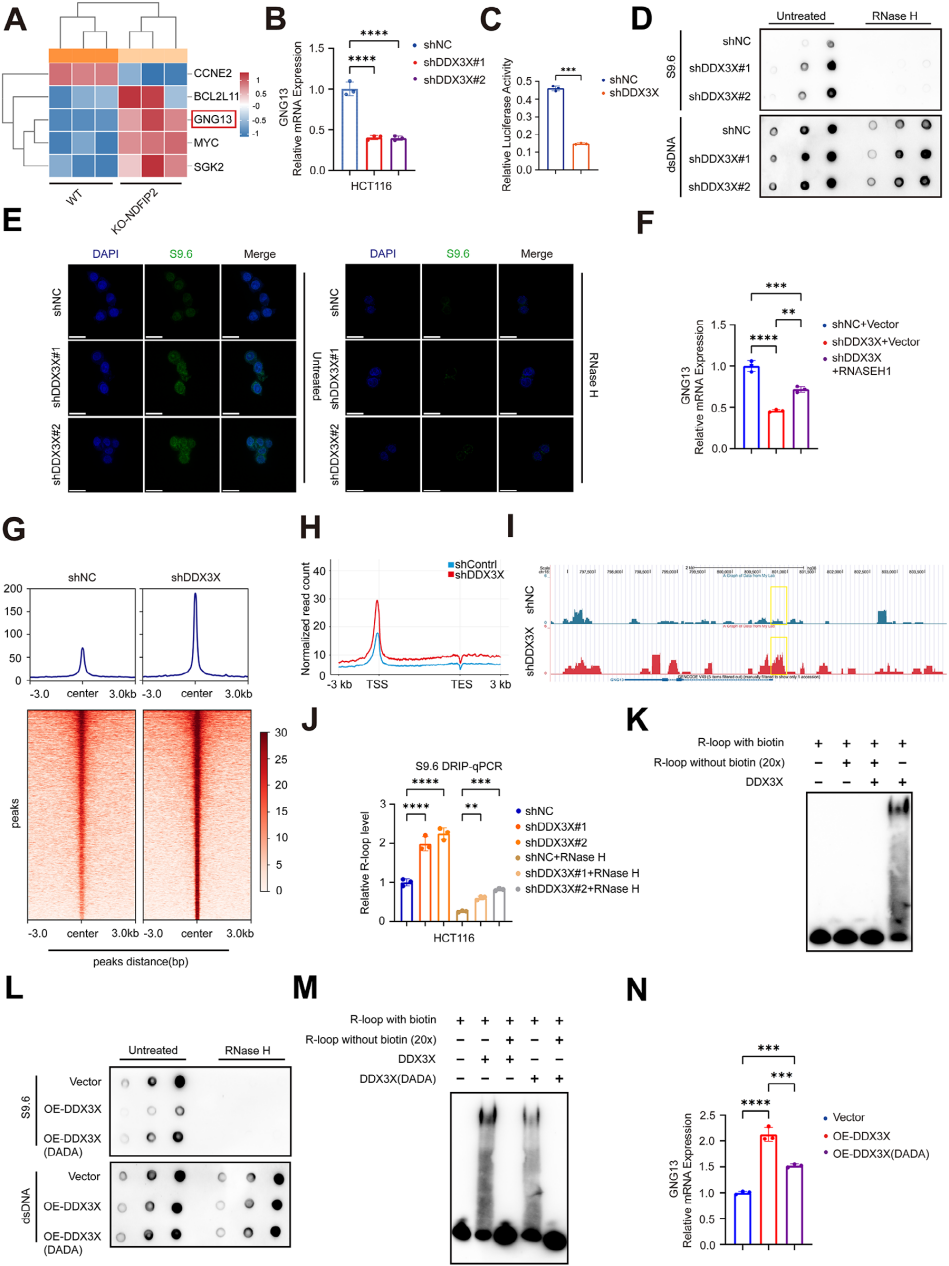
DDX3X resolves promoter‐associated R‐loops to enhance GNG13 transcription. (A) Heatmap showing the top five differentially expressed genes (DEGs), ranked by the magnitude of |log2FC|, within the PI3K/AKT signaling pathway in WT and NDFIP2‐knockout CRC cells, with GNG13 identified as a key candidate for further investigation. (B) qRT‐PCR analysis of GNG13 expression after DDX3X knockdown. Data are presented as mean ± SD (n = 3). Statistical significance was determined using one‐way ANOVA followed by Tukey's multiple comparisons test. ^****^ *p* < 0.0001. (C) Dual‐luciferase assay showing reduced GNG13 promoter activity upon DDX3X knockdown. Data are presented as mean ± SD (n = 3 independent experiments). Statistical significance was determined using a two‐tailed unpaired Student's t‐test. ^***^ *p* < 0.001. (D,E) S9.6 dot blot and immunofluorescence analyses showing increased R‐loop accumulation after DDX3X knockdown, which was abolished by RNase H. Scale bar: 20 µm. (F) qRT‐PCR analysis showing that RNASEH1 partially rescued GNG13 downregulation induced by DDX3X silencing. Data are presented as mean ± SD (n = 3). Statistical significance was determined using one‐way ANOVA followed by Tukey's multiple comparisons test. ^**^ *p* < 0.01, ^***^ *p* < 0.001,^****^ *p* < 0.0001. (G,H) Genome‐wide profiling of S9.6 signals in control and DDX3X‐knockdown cells. (I) Genome browser view showing increased S9.6 signal at the GNG13 locus after DDX3X knockdown. (J) S9.6 DRIP‐qPCR showing increased R‐loop enrichment at the GNG13 promoter after DDX3X knockdown. Data are presented as mean ± SD (n = 3). Statistical significance was determined using one‐way ANOVA followed by Tukey's multiple comparisons test. ^**^ *p* < 0.01, ^***^ *p* < 0.001,^****^ *p* < 0.0001. (K) EMSA showing direct binding of DDX3X to the biotin‐labeled GNG13 promoter‐derived R‐loop probe. (L) S9.6 dot blot showing reduced R‐loop abundance upon DDX3X overexpression, whereas DDX3X(DADA) showed a weaker effect. (M) EMSA comparing the binding of WT DDX3X and DDX3X(DADA) to the R‐loop probe. (N) qRT‐PCR analysis of GNG13 expression in cells expressing WT DDX3X or DDX3X(DADA). All experiments shown in panels A‐N were performed using HCT116 cells. Data are presented as mean ± SD (n = 3). Statistical significance was determined using one‐way ANOVA followed by Tukey's multiple comparisons test. ^***^ *p* < 0.001,^****^ *p* < 0.0001.

Initially, bioinformatic analysis using the TIMER 3.0 database revealed a significant negative correlation between NDFIP2 and GNG13 expression in CRC (Figure S7A). Besides, qPCR analysis revealed that GNG13 expression was markedly reduced upon DDX3X knockdown and significantly increased following DDX3X overexpression (Figure 6B and Figure S7B–D). Functionally, GNG13 silencing further suppressed PI3K/AKT signaling in DDX3X‐deficient cells and attenuated pathway activation induced by NDFIP2 loss (Figure S7E), suggesting that the NDFIP2‐DDX3X axis regulates PI3K/AKT signaling through GNG13. Consistent with these findings, GNG13 knockdown substantially reversed the NDFIP2 depletion‐induced alterations in markers associated with EMT and stemness, as evidenced by reduced N‐cadherin, CD133, and OCT4 expression and restored E‐cadherin expression (Figure S7F). Moreover, GNG13 silencing markedly attenuated the enhanced migratory and invasive capacities of NDFIP2‐deficient CRC cells (Figure S7G,H) and significantly reduced their increased sphere‐forming capacity (Figure S7I,J). Collectively, these results identify GNG13 as a critical downstream mediator of the NDFIP2‐DDX3X axis in regulating metastatic and stemness‐associated phenotypes in CRC cells. To further elucidate the mechanism by which DDX3X regulates GNG13, dual‐luciferase reporter assays demonstrated that DDX3X enhanced GNG13 promoter activity (Figure 6C and Figure S8A), suggesting that DDX3X positively regulates GNG13 at the transcriptional level.

Given the established role of DDX3X as an RNA helicase in maintaining R‐loop homeostasis, which is closely associated with transcriptional regulation [34], we next investigated whether this function underlies its transcriptional regulation of GNG13. Dot blot and immunofluorescence analyses showed that DDX3X depletion led to a marked accumulation of R‐loops, whereas its overexpression reduced R‐loop levels; these signals were abolished by RNase H treatment, confirming its specificity on R‐loop (Figure 6D,E and Figure S8B–E). RNASEH1 overexpression rescued the reduction in GNG13 transcription caused by DDX3X knockdown (Figure 6F), indicating that R‐loop resolution is required for DDX3X‐mediated transcriptional activation.

To further define this mechanism, we performed R‐loop cleavage under targets and tagmentation (CUT&Tag) sequencing, which revealed a global increase in R‐loop abundance upon DDX3X depletion [35], with prominent enrichment at gene promoters (Figure 6G,H). At the GNG13 locus, DDX3X knockdown significantly elevated R‐loop levels across multiple regions, with the most pronounced accumulation at the promoter (Figure 6I). High‐resolution mapping identified a 532‐bp region upstream of the transcription start site as a hotspot of R‐loop enrichment, particularly within the −246 to −177 bp interval (Figure S8F,G). DNA‐RNA immunoprecipitation (DRIP)‐qPCR confirmed that R‐loop levels in this region were increased upon DDX3X knockdown and reduced by DDX3X overexpression, and were abolished by RNase H treatment (Figure 6J and Figure S8H–J).

Electrophoretic mobility shift assays demonstrated that DDX3X directly binds to R‐loop structures within the GNG13 promoter (Figure 6K). To determine whether its helicase activity is required, we generated a helicase‐deficient mutant, DDX3X(DADA). Dot blot analysis showed that DDX3X overexpression markedly reduced global R‐loop levels, whereas DDX3X(DADA) had no significant effect. EMSA confirmed comparable R‐loop‐binding efficiency between DDX3X and DDX3X(DADA). However, although both constructs increased GNG13 mRNA expression, DDX3X(DADA) exerted a significantly weaker effect than wild‐type DDX3X (Figure 6L–N).

Collectively, these findings support a model in which DDX3X promotes GNG13 transcription, at least in part, by resolving promoter‐associated R‐loops, thereby facilitating transcriptional activation of the PI3K/AKT pathway [36].

### 3.6 DDX3X Promotes GNG13 Transcription by Facilitating CTCF Recruitment to its Promoter

In addition to its regulation of R‐loops, DDX3X has also been reported to participate in transcriptional regulation through interaction with specific proteins [37]. We hypothesized that DDX3X may function as a co‐regulator that facilitates the recruitment of transcription factors to the GNG13 promoter. To test this hypothesis, we integrated 678 DDX3X‐interacting proteins from the BioGRID database and found that only two transcription factors, CTCF and MYC, were predicted to bind the GNG13 promoter based on Cistrome DB and JASPAR analyses (Figure 7A).

**FIGURE 7:**
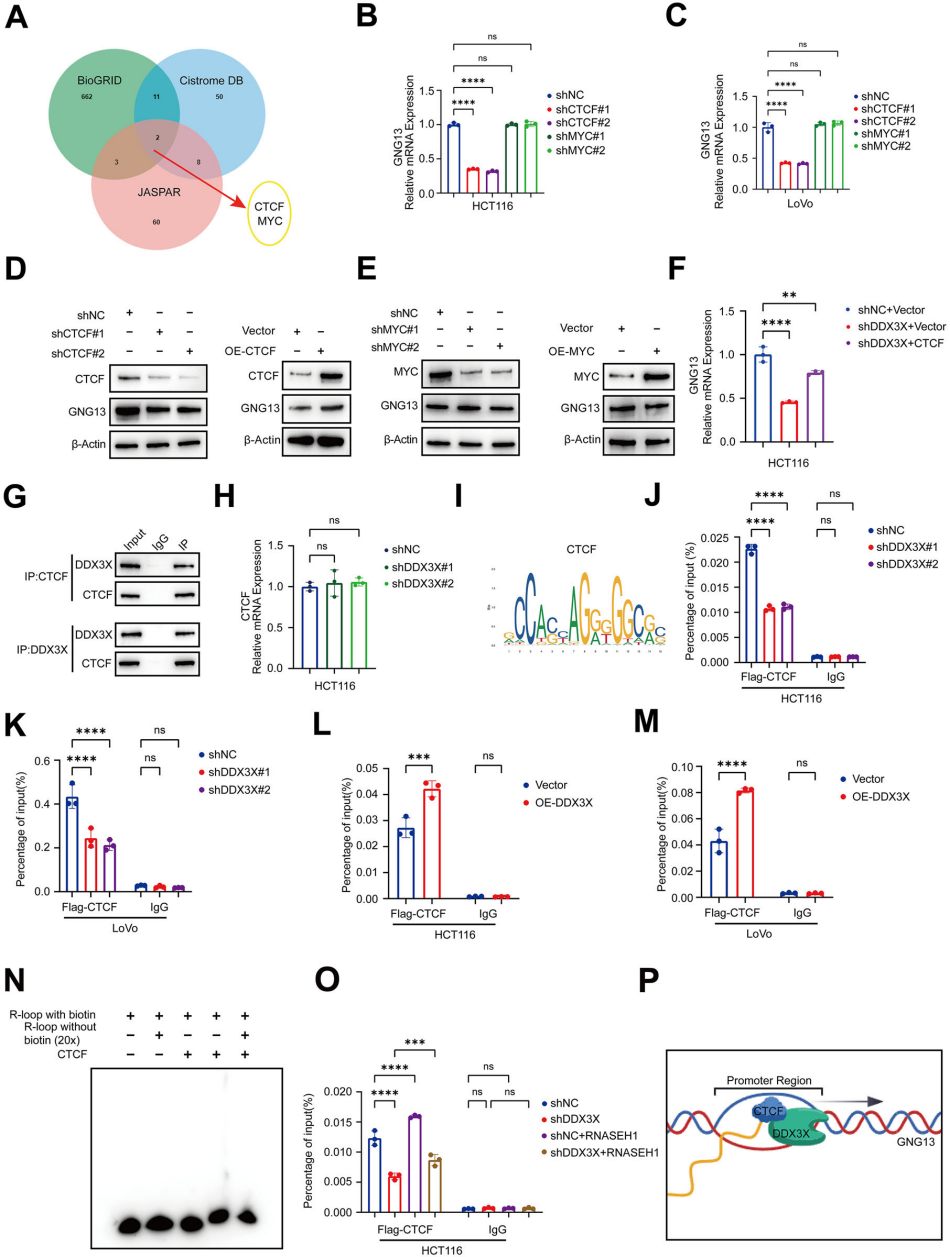
DDX3X promotes GNG13 transcription by facilitating CTCF recruitment to its promoter. (A) Integrated analysis of BioGRID, Cistrome DB, and JASPAR identifying candidate transcription factors potentially involved in DDX3X‐mediated regulation of GNG13. (B,C) qRT‐PCR analysis of GNG13 expression after CTCF or MYC knockdown in HCT116 and LoVo cells. Data are presented as mean ± SD (n = 3). Statistical significance was determined using one‐way ANOVA followed by Dunnett's multiple comparisons test. ns, not significant; ^****^ *p* < 0.0001. (D,E) Immunoblot analysis of GNG13 expression following CTCF or MYC knockdown or overexpression. (F) qRT‐PCR analysis showing that CTCF overexpression partially rescued GNG13 downregulation induced by DDX3X knockdown. Data are presented as mean ± SD (n = 3). Statistical significance was determined using one‐way ANOVA followed by Tukey's multiple comparisons test. ^**^ *p* < 0.01, ^****^ *p* < 0.0001. (G) Exogenous co‐IP assays showing the interaction between DDX3X and CTCF. (H) qRT‐PCR analysis of CTCF mRNA expression after DDX3X knockdown in HCT116 cells. Data are presented as mean ± SD (n = 3). Statistical significance was determined using one‐way ANOVA followed by Dunnett's multiple comparisons test. ns, not significant. (I) Predicted CTCF‐binding motif within the GNG13 promoter. (J,K) ChIP‐qPCR analysis showing that DDX3X knockdown reduced CTCF enrichment at the GNG13 promoter in HCT116 and LoVo cells. Data are presented as mean ± SD (n = 3 independent experiments). Statistical significance was determined using two‐way ANOVA followed by Šídák's multiple comparisons test. ns, not significant; ^****^ *p* < 0.0001. (L,M) ChIP‐qPCR analysis showing that DDX3X overexpression increased CTCF enrichment at the indicated GNG13 promoter regions in HCT116 and LoVo cells. Data are presented as mean ± SD (n = 3). Statistical significance was determined using two‐way ANOVA followed by Šídák's multiple comparisons test. ns, not significant; ^***^ *p* < 0.001,^****^ *p* < 0.0001. (N) EMSA assay showing the interaction of CTCF with the indicated GNG13 promoter‐derived R‐loop probe. (O) ChIP‐qPCR analysis of CTCF enrichment at the GNG13 promoter in DDX3X‐depleted cells treated with or without RNASEH1 overexpression. Data are presented as mean ± SD (n = 3). Statistical significance was determined using two‐way ANOVA followed by Tukey's multiple comparisons test. ns, not significant; ^***^ *p* < 0.001,^****^ *p* < 0.0001. (P) Schematic model illustrating that DDX3X facilitates CTCF recruitment to the GNG13 promoter, thereby promoting GNG13 transcription.

Functional screening revealed that CTCF knockdown markedly reduced GNG13 expression at both mRNA and protein levels, whereas CTCF overexpression increased its expression. In contrast, MYC manipulation had no significant effect (Figure 7B–E and Figure S9A,B), indicating that CTCF, but not MYC, drives GNG13 transcription [38]. Notably, CTCF overexpression largely rescued the reduction in GNG13 expression caused by DDX3X knockdown (Figure 7F), suggesting that DDX3X regulates GNG13 in a CTCF‐dependent manner.

Co‐immunoprecipitation assays confirmed a physical interaction between DDX3X and CTCF (Figure 7G and Figure S9C). DDX3X depletion did not alter CTCF expression at either the mRNA or protein level (Figure 7H and Figure S9D,E), indicating that DDX3X modulates CTCF recruitment rather than its abundance.

Motif analysis using JASPAR identified a putative CTCF‐binding site within the GNG13 promoter (chr16:801339‐801354) (Figure 7I). Chromatin immunoprecipitation (ChIP)‐qPCR assays further demonstrated that DDX3X knockdown significantly reduced CTCF occupancy at the GNG13 promoter, whereas DDX3X overexpression enhanced CTCF enrichment (Figure 7J–M). Notably, EMSA showed that CTCF does not directly bind to R‐loop structures within the GNG13 promoter (Figure 7N), suggesting that its transcriptional activity is independent of R‐loop recognition. To further elucidate the interplay between R‐loop dynamics and CTCF recruitment, we performed rescue experiments. Notably, clearance of R‐loops via RNASEH1 overexpression significantly bolstered CTCF binding at the GNG13 promoter. More importantly, the reduction in CTCF occupancy induced by DDX3X deficiency was substantially abrogated by RNASEH1 overexpression (Figure 7O), underscoring that DDX3X‐mediated R‐loop resolution is a prerequisite for efficient CTCF loading and subsequent GNG13 transcription.

To validate the physiological relevance of this regulatory axis, we performed phenotypic rescue experiments. The inhibitory effects of NDFIP2 overexpression on CRC cell migration and invasion were effectively counteracted by concurrent RNASEH1 overexpression (Figure S9F,G).

Collectively, these findings elucidate a pivotal mechanism wherein DDX3X promotes GNG13 transcription by simultaneously resolving promoter‐associated R‐loops and facilitating CTCF recruitment, thereby driving CRC metastasis (Figure 7P).

### 3.7 The iRGD‐Conjugated NDFIP2 Residues 147–220 Peptide Inhibits CRC Metastatic Progression in Vitro and in Vivo

The PY motifs of NDFIP2 are critical for its interaction with DDX3X and its downstream regulatory functions. Domain‐mapping analyses identified a strong interaction between DDX3X and NDFIP2 residues 147–220, a region enriched in PY motifs (Figure 8A). To further define the functional domain, we generated three truncated constructs spanning NDFIP2 (1‐146, 147–220, and 221–336) (Figure 8B). Co‐immunoprecipitation assays demonstrated that the 147–220 fragment was sufficient for DDX3X binding (Figure 8C).

**FIGURE 8:**
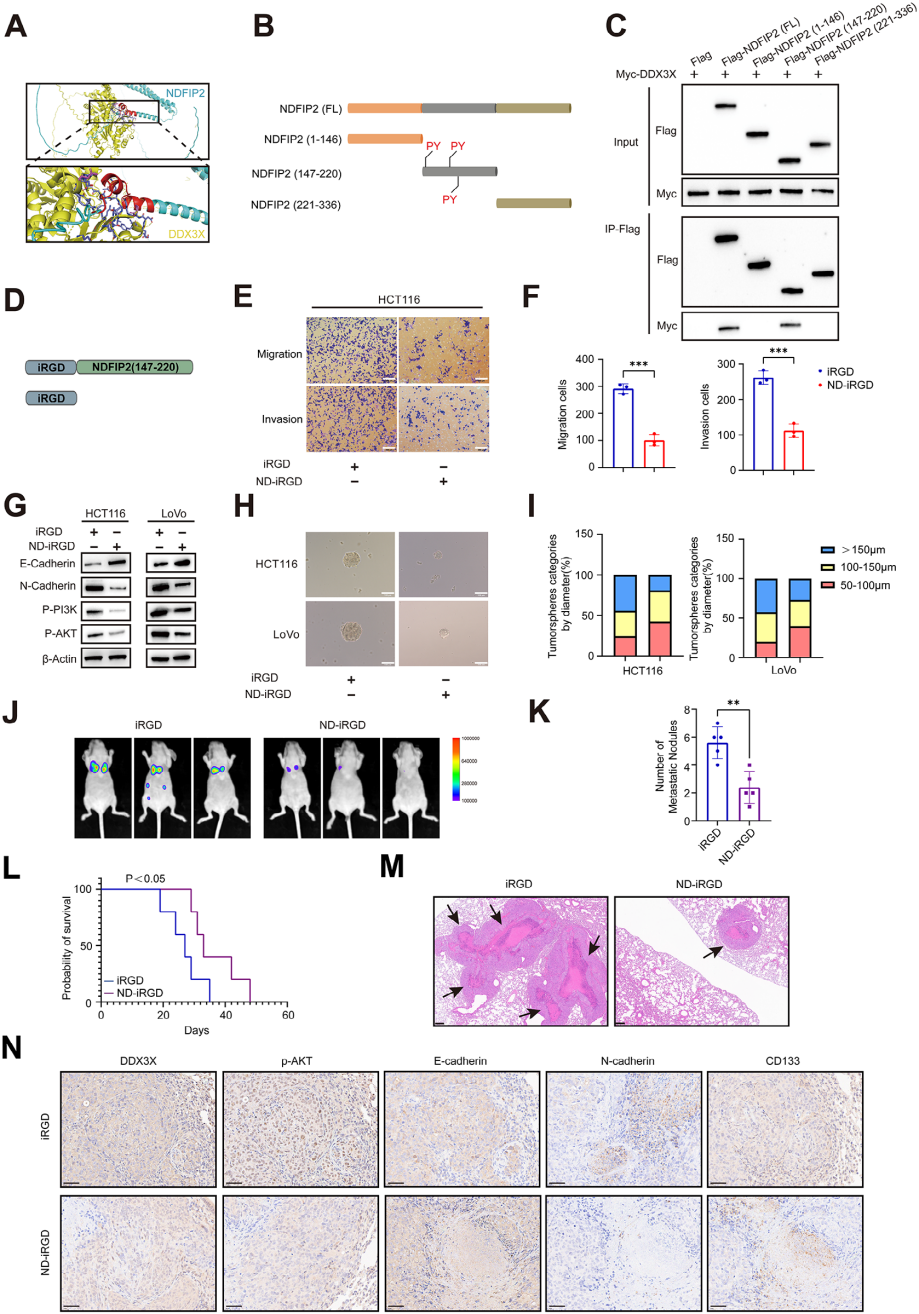
The iRGD‐conjugated NDFIP2 residues 147–220 peptide inhibits CRC metastatic progression in vitro and in vivo. (A) Structural model showing the predicted interaction between NDFIP2 and DDX3X. (B) Schematic of full‐length NDFIP2 and the indicated truncation mutants used for domain‐mapping analysis. (C) Co‐IP analysis showing the interaction of Myc‐DDX3X with full‐length or truncated Flag‐NDFIP2 constructs, identifying the DDX3X‐binding region of NDFIP2. (D) Schematic of the iRGD‐conjugated NDFIP2 peptide (ND‐iRGD) and the iRGD control. (E,F) Transwell migration and invasion assays showing that ND‐iRGD suppressed the migratory and invasive capacities of HCT116 cells. Scale bar: 200 µm. Data are presented as mean ± SD (n = 3 independent experiments). Statistical significance was determined using a two‐tailed unpaired Student's t‐test. ^***^ *p* < 0.001. (G) Immunoblot analysis of EMT markers and PI3K/AKT pathway‐related proteins in HCT116 and LoVo cells treated with iRGD or ND‐iRGD. (H,I) Representative images and quantification of tumorsphere formation showing that ND‐iRGD reduced CRC stemness. Scale bar: 100 µm. Representative images and quantification of HCT116 and LoVo tumorspheres treated with the ND‐iRGD fusion peptide or the iRGD control peptide at 10 µM starting on day 4 and analyzed on day 7. (J) Representative bioluminescence images of lung metastases in mice intravenously injected with HCT116 cells. Beginning 1 week after tumor cell inoculation, mice received intraperitoneal injections of iRGD or the ND‐iRGD fusion peptide at 25 µmol/kg every 3 days for 2 weeks. (K) Quantification of the number of metastatic lung nodules in each group. Data are presented as mean ± SD (n = 5). Statistical significance was determined using a two‐tailed unpaired Student's t‐test. ^**^ *p* < 0.01. (L) Kaplan‐Meier survival analysis of mice in the indicated groups (n = 5). Statistical significance was determined using the log‐rank (Mantel‐Cox) test. P < 0.05 was considered statistically significant. (M) Representative H&E staining of lung sections from the indicated groups. Arrows indicate metastatic lesions. Scale bar: 200 µm. (N) Representative immunohistochemical staining of DDX3X, p‐AKT, E‐cadherin, N‐cadherin, and CD133 in lung metastatic lesions from iRGD‐ or ND‐iRGD‐treated mice. Scale bar: 50 µm. For panels E–G, HCT116 cells were treated with the ND‐iRGD fusion peptide or the iRGD control peptide at 10 µM for 24 h before the indicated analyses.

To explore the therapeutic potential of this functional domain, we conjugated the NDFIP2 (147‐220) fragment to the tumor‐penetrating peptide iRGD [39], generating a fusion peptide termed ND‐iRGD (Figure 8D). In vitro, ND‐iRGD treatment markedly suppressed CRC cell migration and invasion (Figure 8E,F and Figure S10A,B). Consistently, ND‐iRGD increased E‐cadherin expression while reducing N‐cadherin, p‐PI3K, and p‐AKT levels (Figure 8G), indicating inhibition of epithelial‐mesenchymal transition and PI3K/AKT signaling.

In addition, ND‐iRGD significantly impaired cancer stem‐like properties, as evidenced by reduced tumorsphere size and number, along with decreased expression of stemness‐associated markers (Figure 8H,I and Figure S10C,D).

To evaluate the antimetastatic efficacy in vivo, CRC cells were intravenously injected into mice, followed by the intraperitoneal administration of equimolar doses (25 µmol/kg) of either iRGD or ND‐iRGD every 72 h. In vivo fluorescence imaging revealed markedly reduced pulmonary metastatic burden in the ND‐iRGD‐treated group compared with controls (Figure 8J). This was corroborated by a significant decrease in the number of lung metastatic nodules and a prolongation of overall survival (Figure 8K,L). Besides, H&E staining also revealed a significant reduction in both the number and size of lung metastatic lesions in the ND‐iRGD group relative to the iRGD group (Figure 8M). Immunohistochemical analysis further demonstrated reduced expression of DDX3X, p‐AKT, N‐cadherin, and CD133, along with increased E‐cadherin expression, in metastatic lesions from ND‐iRGD‐treated mice (Figure 8N and Figure S10E). Accordingly, we evaluated the role of NDFIP2 in vivo by establishing nude mouse liver metastasis models. The ND‐iRGD group revealed a markedly reduced liver metastatic burden compared with control groups (Figure S10F). Furthermore, this therapeutic effect was corroborated by a significant decrease in the number of liver metastatic nodules (Figure S10G,H). To explore the underlying molecular mechanisms, immunohistochemical (IHC) analysis was performed on the liver metastatic nodules. These results confirmed that ND‐iRGD led to the upregulation of E‐cadherin, and the downregulation of p‐AKT, N‐cadherin, and CD133 (Figure S10I,J). Moreover, the ND‐iRGD group exhibited a significantly prolonged overall survival compared to the iRGD group (Figure S10K).

Mechanistically, GNG13 overexpression substantially reversed the ND‐iRGD‐induced reductions in p‐PI3K, p‐AKT, and N‐cadherin expression, while attenuating the upregulation of E‐cadherin (Figure S11A,B). Consistent with these molecular changes, GNG13 overexpression markedly rescued the inhibitory effects of ND‐iRGD on the migratory and invasive capacities of CRC cells (Figure S11C,D) and restored the sphere‐forming capacity suppressed by ND‐iRGD (Figure S11E,F). Collectively, these rescue experiments demonstrate that the antimetastatic and stemness‐suppressive effects of ND‐iRGD are mediated, at least in part, through inhibition of the GNG13‐PI3K/AKT signaling axis.

Collectively, these results identify the NDFIP2 147–220 region as a critical functional domain that suppresses CRC metastasis and demonstrate its potential as a peptide‐based therapeutic strategy.

## 4 Discussion

Distant metastasis represents the most formidable challenge in the clinical management of CRC, accounting for the vast majority of cancer‐related deaths. Despite advancements in surgical techniques and systemic therapies, the molecular “black box” governing the transition of primary tumor cells into invasive, metastatic entities remains only partially understood [40]. In this study, we identify NDFIP2 as a previously unrecognized metastasis suppressor in CRC and delineate a sophisticated regulatory network that bridges post‐translational modification, epigenetic structural dynamics, and oncogenic signaling. Our findings not only expand the functional repertoire of NDFIP2 but also provide a conceptual shift in how we perceive R‐loop homeostasis in the context of tumor progression.

A pivotal observation of our study is the epithelial‐intrinsic role of NDFIP2. Through single‐cell transcriptomic profiling and clinical validation, we demonstrated that NDFIP2 is predominantly downregulated in the tumor epithelial compartment of metastatic CRC tissues. While NDFIP2 is also expressed in CAFs and T cells, its expression levels in these stromal populations showed no significant correlation with metastatic burden or clinical outcomes. This specificity is crucial from a therapeutic perspective; whereas many current anti‐metastatic strategies target the broad tumor microenvironment (TME) and often suffer from systemic off‐target effects, NDFIP2 represents a high‐fidelity epithelial “brake”. The loss of this brake triggers a cascade of EMT and stemness acquisition, which we have shown is primarily mediated by the hyperactivation of the PI3K/AKT pathway.

The mechanistic heart of this study lies in the NDFIP2‐mediated proteasomal degradation of the RNA helicase DDX3X. Previous studies have shown that DDX3X is involved in the regulation of mRNA metabolism and translational programs [41]. We have elucidated that NDFIP2 does not directly possess E3 ligase activity but functions as a molecular matchmaker. Specifically, NDFIP2 utilizes its PY motifs to recruit the HECT‐type E3 ligase NEDD4. The structural precision of this interaction is remarkable; the PY motif adopts a unique, left‐handed polyproline type II (PPII) helix that provides a stable docking surface for the WW domains of NEDD4 [42]. This allosteric recruitment facilitates the targeted K48‐linked polyubiquitination of DDX3X, specifically at the K264 residue. Our domain‐mapping and site‐directed mutagenesis experiments confirm that this specific ubiquitination event is the “molecular switch” that dictates DDX3X stability. This finding adds a new layer of complexity to the regulation of DEAD‐box helicases, suggesting that their abundance in cancer cells is not merely a reflection of transcriptional output but is tightly governed by specialized ubiquitin‐proteasome adaptors like NDFIP2.

Previous work by Eide et al. demonstrated that NEDD4 is overexpressed in CRC and promotes colon cancer cell growth, suggesting an oncogenic role for NEDD4 [43]. Conversely, intestinal deletion of Nedd4 accelerated tumor growth in a murine model of CRC, suggesting that NEDD4 may also exert tumor‐suppressive effects in certain contexts [44]. These apparently divergent findings indicate that the biological function of NEDD4 is highly dependent on the genetic background, regulatory adaptors, and recruited substrates. Notably, the study by Eide et al. primarily focused on tumor cell growth, whereas our study addresses metastasis. In our model, the adaptor protein NDFIP2 recruits NEDD4 to DDX3X, thereby promoting DDX3X ubiquitination and degradation and ultimately suppressing CRC metastasis. Thus, NEDD4 may exert oncogenic or tumor‐suppressive effects in CRC depending on the phenotypic context and the specific substrates it targets.

A defining innovation of our research is the characterization of R‐loops as active “transcriptional roadblocks” rather than mere byproducts of transcriptional stress. Conventionally, unresolved R‐loops are viewed as threats to genomic integrity, often precipitating DNA double‐strand breaks and replication stress [45]. While previous studies have demonstrated that physiological R‐loops can sustain transcriptional activity by shielding gene promoters from DNA methylation [46], their aberrant accumulation at promoter regions can profoundly repress the transcription of associated genes [45]. Building upon this duality, our data support an emerging paradigm wherein tumor cells actively exploit R‐loop resolution to “unlock” oncogenic transcription. We found that in the absence of NDFIP2, an aberrantly stabilized DDX3X specifically targets the GNG13 promoter. By resolving the R‐loops naturally enriched at this precise locus, DDX3X alleviates local steric hindrance, thereby orchestrating the recruitment of the architectural protein CTCF.

This DDX3X‐CTCF interplay represents a novel epigenetic mechanism in CRC metastasis. CTCF recruitment is often dependent on specific chromatin landscapes and DNA methylation states [47]. Our results suggest that R‐loop resolution by DDX3X creates a “permissive” chromatin state at the GNG13 promoter, allowing CTCF to bind and activate transcription. Given that Gγ13 [48] is a potent upstream activator of the PI3K/AKT cascade [49], this axis constitutes a self‐amplifying feed‐forward loop in metastatic cells: R‐loop resolution leads to GNG13 expression, which drives PI3K/AKT signaling [50], further promoting EMT and invasion. Conversely, the presence of NDFIP2 ensures that DDX3X levels remain low, thereby maintaining the R‐loop “roadblock” and keeping the GNG13‐PI3K axis in a quiescent state. This duality underscores the potential of targeting R‐loop‐resolving helicases as a strategy to reinstate transcriptional silencing of oncogenic networks.

Furthermore, we bridged these mechanistic insights with a translatable therapeutic design. Although small‐molecule inhibitors of the PI3K/AKT pathway, such as ipatasertib, have shown promise, their clinical utility is often hampered by narrow therapeutic windows [51]. To overcome these hurdles, we engineered a biomimetic ND‐iRGD fusion peptide. The inclusion of the iRGD motif is strategic; it exploits the “C‐end Rule” (CendR) pathway, where initial binding to αvβ3/β5 integrins on tumor vasculature is followed by a proteolytic cleavage that exposes a C‐terminal motif (RGDK/R). This motif then engages Neuropilin‐1 (NRP‐1) to trigger massive transcytosis and tissue penetration [52]. The translational feasibility of this approach is strongly supported by recent clinical evidence, wherein combining iRGD (CEND‐1) with standard gemcitabine plus nab‐paclitaxel therapy has demonstrated remarkable preliminary efficacy in patients with advanced metastatic pancreatic cancer. Compared to the standard regimen alone, this combination significantly improved the objective response rate (ORR) and prolonged progression‐free survival (PFS) [53].

Crucially, our preclinical data demonstrate that this peptide effectively mimics the endogenous function of NDFIP2, driving DDX3X degradation and restoring the R‐loop‐mediated silencing of GNG13. The striking reduction in pulmonary metastatic nodules and the significant prolongation of survival in our murine models provide a strong rationale for the clinical development of peptide‐based “R‐loop stabilizers”. Unlike broad inhibitors, this approach targets a specific molecular vulnerability identified through deep mechanistic dissection, potentially offering a more potent and less toxic alternative for patients with metastatic CRC.

This study has several limitations. First, it remains unclear whether other RNA helicases are similarly regulated by the NDFIP2–NEDD4 complex or whether DDX3X represents a selectively regulated RNA helicase substrate in CRC. Second, although we focused on the GNG13 promoter, DDX3X‐mediated R‐loop resolution may have broader effects on the metastatic transcriptome. Future studies integrating the R‐loop CUT&Tag maps generated in the present study with nascent RNA sequencing will help define the broader transcriptional consequences of DDX3X‐mediated R‐loop remodeling. Finally, although the in vivo findings were statistically significant, the relatively small animal cohorts limited their robustness and generalizability. Larger animal cohorts will therefore be employed in future translational preclinical studies to further validate the therapeutic potential of this strategy.

## 5 Conclusion

In conclusion, NDFIP2 acts as a critical suppressor of CRC metastasis. Mechanistically, NDFIP2 recruits NEDD4 to drive DDX3X degradation. This promotes R‐loop accumulation at the GNG13 promoter and blocks CTCF binding, effectively silencing GNG13 and restraining oncogenic PI3K/AKT signaling. Furthermore, our engineered ND‐iRGD fusion peptide showed remarkable efficacy in mitigating distant metastasis and prolonging murine survival. These findings highlight R‐loop‐mediated oncogenic regulation and present the ND‐iRGD peptide as a promising translatable therapeutic for metastatic CRC.

## Author Contributions

YZ and EJZ conceived and designed the experiments. YFL, SX and JXS analyzed the data. HL participated in data acquisition and interpretation. PBZ, ZQR, and CZ provided revision guidance and experimental design support. JXS, XZZ, WN, and SG performed additional experiments and data validation. XL contributed reagents, materials, and analysis tools. YZ and EJZ wrote the main manuscript. All authors read and approved the final manuscript.

## Ethics Statement

All animal experiments were reviewed and approved by the Laboratory Animal Ethics Committee of Xuzhou Medical University (approval no. 202503T034) and conducted in accordance with the institutional guidelines for the care and use of laboratory animals. The use of human tissue samples was reviewed and approved by the Ethics Committee of the Affiliated Hospital of Xuzhou Medical University (approval no. XYFY2025‐KL386‐01), and all procedures involving human participants were conducted in accordance with the Declaration of Helsinki. Written informed consent was obtained from all participants prior to surgery.

## Consent for Publication

All authors give consent for the publication of the manuscript.

## Conflicts of Interest

The authors declare no conflicts of interest.

## Supporting information




**Supporting File 2**: advs78466‐sup‐0001‐SuppMat.docx.

**Supporting File 1**: advs78466‐sup‐0002‐SuppMat.xlsx.

## Data Availability

The data that support the findings of this study are available from the corresponding author upon reasonable request.
