# Supplementary material for: NDFIP2 Suppresses Colorectal Cancer Metastasis Through DDX3X‐Dependent R‐Loop Remodeling and GNG13 Transcriptional Activation
Source: Adv Sci (Weinh). 2026 Oct 31:e78466. Online ahead of print. doi: 10.1002/advs.78466 (PMC13723715; doi:10.1002/advs.78466)
Supplement: Supplementary file 1 [file ADVS-9999-e78466-s001.docx]

**NDFIP2 Suppresses Colorectal Cancer Metastasis through DDX3X-Dependent R-loop Remodeling and GNG13 Transcriptional Activation**

Enjian Zhang, Yanfang Li et al.

Corresponding author: Xin Liu, Yi Zhang.

**The file includes:**

Supplementary materials

Supplementary Figures

Figs. S1 to S11

**Supplemental Materials**

**List of antibodies used in the paper**

| Species Citation | Antigen | Clone# | Company (Cat#) | RRID |
| --- | --- | --- | --- | --- |
| Rabbit polyclonal | NDFIP2 |  | Invitrogen (PA5-100882) | AB_2850378 |
| Rabbit polyclonal | DDX3X |  | ABclonal (A5637) | AB_2766397 |
| Rabbit polyclonal | GNG13 |  | Abmart (PA3966) | AB_3754818 |
| Rabbit polyclonal | CTCF |  | Abcam (ab70303) | AB_1209546 |
| Rabbit polyclonal | E-cadherin |  | Proteintech (20874-1-AP) | AB_10697811 |
| Rabbit polyclonal | N-cadherin |  | Proteintech (22018-1-AP) | AB_2813891 |
| Rabbit polyclonal | CD133 |  | Abcam (ab19898) | AB_470302 |
| Mouse monoclonal | CD44 | 156-3C11 | Cell Signaling Technology (#3570) | AB_2076465 |
| Rabbit polyclonal | OCT4 |  | Proteintech (11263-1-AP) | AB_2167545 |
| Rabbit polyclonal | SOX2 |  | Proteintech (11064-1-AP) | AB_2195801 |
| Mouse monoclonal | Flag-Tag | 8H6A10 | Proteintech (66008-4-Ig) | AB_2918475 |
| Rabbit monoclonal | β-Actin | ARC5115-01 | ABclonal (AC026) | AB_2768234 |
| Rabbit polyclonal | PI3K |  | Proteintech (20584-1-AP) | AB_10734439 |
| Rabbit polyclonal | p-PI3K |  | Cell Signaling Technology (#4228) | AB_659940 |
| Rabbit polyclonal | AKT |  | Proteintech (10176-2-AP) | AB_2224574 |
| Mouse monoclonal | p-AKT | 1C10B8 | Proteintech (66444-1-Ig) | AB_2782958 |
| Rabbit polyclonal | PRAS40 |  | Proteintech (21097-1-AP) | AB_2878811 |
| Rabbit Monoclonal | p-PRAS40 | D4D2 | Cell Signaling Technology (#13175) | AB_2798140 |
| Mouse monoclonal | Myc-Tag | 1A5A2 | Proteintech (60003-2-Ig) | AB_2734122 |
| Rabbit polyclonal | NEDD4 |  | Proteintech (21698-1-AP) | AB_10858626 |
| Mouse monoclonal | S9.6 |  | Kerafast (ENH001) | AB_2687463 |
| Mouse monoclonal | HA-Tag | 1F5C6 | Proteintech (66006-2-Ig) | AB_2881490 |
| Mouse monoclonal | dsDNA | 35I9 DNA | Abcam (ab27156) | AB_470907 |
| Mouse monoclonal | β-Tubulin | A1-A4 | HUABIO (M1305-2) | AB_3073058 |

**List of primers and oligos used in the paper**

| Usage | Name | Forward(5’-3’) | Reverse(5’-3’) |
| --- | --- | --- | --- |
| qRT-PCR | NDFIP2 | CTGGCATTTTTCACTGGAAGGT | TGTCGGGTTGATGCAGTCTC |
|  | DDX3X | GTGGAAGTGGATCAAGGGGA | TGATTTGTCACACCAGCGAC |
|  | GNG13 | CCGCTGTCACCTTTTCAAGC | CCGTCCTCGATCCACTTCAG |
|  | CTCF | CAGCCACGGAGAGGTACTCG | GGCGTAATCGCACATGGAAC |
| ChIP-qPCR | GNG13-  Promoter | CAGCCCAGAAACTAAGGTGC | GCTAGACGTCTCCCTGATCC |
| DRIP-qPCR | GNG13-  Promoter  (-118,0) | ACAGGCTATCTCTGGGGTCA | GATGTTCCGGGAGTCCGC |
|  | GNG13-  Promoter  (-246,-177) | CACCCCCTCAAGTGGAAGGA | GGCTGGTCCATTTTCCCTGA |
|  | GNG13-  Promoter  (-390,-303) | ACATGGCCCACACAGGATG | CTTTGCCCTCTGAAGCCTCT |
|  | GNG13-  Promoter  (-532,-401) | CTGAAAAGCACGGCACTCTC | GGGATGTGAGCCTCTTACTGC |
| R-loop  EMSA | DNA oligo-1 | 5′GCCCCATCCCCACTCCTCCCGGCTGGCCCAGGACACTGGAGGGGGAACCCACCCCCTCAAGTGGAAGGAGACCTCTCCACCAGGGCCCAAGCTGGCCCC | |
|  | DNA oligo-2 | 5′GGGGCCAGCTTGGGCCCTGGTGGAGAGGTCTCCTTCCACTTGAGGGGGTGGGTTCCCCCTCCAGTGTCCTGGGCCAGCCGGGAGGAGTGGGGATGGGGC | |
|  | RNA oligo | (Biotin)5′CCAGUGUCCUGGGCCAGCCGGGAGGAG | |

**shRNA used in the paper**

| Name | Target | shRNA | Loop |
| --- | --- | --- | --- |
| shDDX3X#1 | DDX3X | 5’CGCTTGGAACAGGAACTCTTT | CTCGAG |
| shDDX3X#2 |  | 5’CGTAGAATAGTCGAACAAGAT |  |
| shMYC#1 | MYC | 5’CAGGAACTATGACCTCGACTA |  |
| shMYC#2 |  | 5’GAATGTCAAGAGGCGAACACA |  |
| shCTCF#1 | CTCF | 5’GCTGTGTTTCATGAGCGCTAT |  |
| shCTCF#2 |  | 5’GCCTCTTTCTTGGCAAAGTTT |  |

**sgRNA used in the paper**

| Name | Target | sgRNA |
| --- | --- | --- |
| NDFIP2-KO1 | NDFIP2 | 5’AGCATGCTCAATAGCGCGCG |
| NDFIP2-KO2 |  | 5’TCGACGACGTCGTCGCCGCA |

**Supplementary Figures**

**Supplementary Figure S1. Low NDFIP2 expression is closely associated with CRC metastasis and a poor prognosis**

**(A)** Relative NDFIP2 mRNA expression in T2N0M0 and T2N0M1 CRC tissues. Data are presented as mean ± SD (n = 6 patients per group). Statistical significance was determined using a two-tailed unpaired Welch's t-test. **P < 0.01. **(B)** Immunoblot analysis of NDFIP2 expression in T2N0M0 and T2N0M1 CRC tissues, with β-Actin as a loading control. **(C)** Kaplan-Meier analyses of RFS and OS stratified by NDFIP2 expression in CRC patients. **(D)** Representative multiplex immunofluorescence images showing the expression of NDFIP2, E-cadherin, and N-cadherin in T2N0M0 and T2N0M1 CRC tissues (n = 6 patients per group). Nuclei were counterstained with DAPI. Scale bars: 100 μm. **(E)** Quantification of the percentages of cells positive for NDFIP2, E-cadherin, and N-cadherin in T2N0M0 and T2N0M1 CRC tissues (n = 6 patients per group). Data are presented as median with interquartile range. Statistical significance was determined using a two-tailed Mann-Whitney U test. **P < 0.01.

**Supplementary Figure S2. NDFIP2 suppresses CRC migration, invasion, and stemness**

**(A)** Immunoblot validation of NDFIP2 knockout efficiency in independently generated LoVo cell clones. Tubulin served as a loading control. **(B, C)** Representative images and quantification of Transwell migration and invasion assays in LoVo cells with wild-type control (WT) or NDFIP2 knockout (KO1-NDFIP2 and KO2-NDFIP2). Scale bar: 200 μm. Data are presented as mean ± SD (n = 3). Statistical significance was determined using one-way ANOVA followed by Tukey's multiple comparisons test. ****P < 0.0001. **(D)** Representative images and quantification of Transwell migration and invasion assays in LoVo cells transfected with empty vector or NDFIP2 overexpression construct (OE-NDFIP2). Scale bar: 200 μm. Data are presented as mean ± SD (n = 3). Statistical significance was determined using a two-tailed unpaired Student's t-test. ****P < 0.0001. **(E)** Quantification of tumorsphere diameter distribution in HCT116 and LoVo cells with WT, KO1-NDFIP2, or KO2-NDFIP2. Tumorspheres were classified according to diameter as 50-100 μm, 100-150 μm, or >150 μm. **(F)** Quantification of tumorsphere numbers in HCT116 and LoVo cells with WT, KO1-NDFIP2, or KO2-NDFIP2. Data are presented as mean ± SD (n = 3). Statistical significance was determined using one-way ANOVA followed by Tukey's multiple comparisons test. **P < 0.01, ***P < 0.001,****P < 0.0001. **(G)** Quantification of tumorsphere diameter distribution in HCT116 and LoVo cells transfected with empty vector or OE-NDFIP2. Tumorspheres were classified according to diameter as 50-100 μm, 100-150 μm, or >150 μm. **(H)** Quantification of tumorsphere numbers in HCT116 and LoVo cells transfected with empty vector or OE-NDFIP2. Data are presented as mean ± SD (n = 3). Statistical significance was determined using a two-tailed unpaired Student's t-test. **P < 0.01.

**Supplementary Figure S3. NDFIP2 suppresses colorectal cancer metastasis by inhibiting PI3K/AKT signaling**

**(A, B)** Immunoblot analysis of stemness-associated markers, including CD133, CD44, and OCT4, in HCT116 **(A)** and LoVo **(B)** cells with wild-type control (WT) or NDFIP2 knockout (KO1-NDFIP2 and KO2-NDFIP2). β-Actin served as a loading control. **(C, D)** Immunoblot analysis of CD44, CD133, and OCT4 in HCT116 **(C)** and LoVo **(D)** cells transfected with empty vector or NDFIP2 overexpression construct (OE-NDFIP2). β-Actin served as a loading control. **(E, F)** Immunoblot analysis of PI3K, phospho-PI3K (p-PI3K), AKT, and phospho-AKT (p-AKT) in HCT116 **(E)** and LoVo **(F)** cells transfected with empty vector or OE-NDFIP2. β-Actin served as a loading control. **(G)** CCK-8 analysis of HCT116 and LoVo cells treated with DMSO or ipatasertib for the indicated durations. Cell viability was determined by measuring the absorbance at 450 nm. Data are presented as mean ± SD (n = 3). Statistical significance was determined using two-way ANOVA followed by Šídák's multiple comparisons test. ****P < 0.0001. **(H, I)** Representative images and quantification of Transwell migration and invasion assays using HCT116 **(H)** and LoVo **(I)** cells treated with DMSO or ipatasertib. Scale bars: 200 μm. Data are presented as mean ± SD (n = 3). Statistical significance was determined using a two-tailed unpaired Student's t-test. ****P < 0.0001. **(J)** Representative in vivo bioluminescence images of mice bearing metastatic tumors treated with DMSO or ipatasertib. **(K)** Quantification of metastatic nodules in the DMSO and ipatasertib groups (n = 5). Data are presented as mean ± SD. Statistical significance was determined using a two-tailed unpaired Student’s t-test. ***P < 0.001. **(L-N)** Representative images **(L)** and quantification of Transwell migration **(M)** and invasion **(N)** assays in LoVo cells with WT, KO1-NDFIP2, or KO2-NDFIP2 treated with DMSO or the AKT inhibitor ipatasertib. Scale bar: 200 μm. Data are presented as mean ± SD (n = 3). Statistical significance was determined using one-way ANOVA followed by Tukey's multiple comparisons test. ****P < 0.0001. **(O)** Quantification of tumorsphere numbers in HCT116 and LoVo cells with WT, KO1-NDFIP2, or KO2-NDFIP2 treated with DMSO or ipatasertib. Data are presented as mean ± SD (n = 3). Statistical significance was determined using one-way ANOVA followed by Tukey's multiple comparisons test. ****P < 0.0001. **(P)** Immunoblot analysis of NDFIP2, E-cadherin, N-cadherin, PRAS40, p-PRAS40, and stemness-associated markers (CD133, CD44, OCT4, and SOX2) in HCT116 cells under the indicated conditions. β-Actin served as a loading control.

**Supplementary Figure S4. NDFIP2 suppresses colorectal cancer metastasis by inhibiting PI3K/AKT signaling**

**(A, B)** Quantification of IHC staining for NDFIP2, p-PRAS40, E-cadherin, N-cadherin, and CD133 using H-scores in metastatic tumor tissues from the indicated groups. Statistical significance was determined using one-way ANOVA followed by Tukey's multiple comparisons test. ns, not significant; ****P < 0.0001.

**Supplementary Figure S5. DDX3X knockdown reverses NDFIP2 deficiency-induced CRC cell migration, invasion, and stemness**

**(A)** Endogenous co-IP showing the interaction between NDFIP2 and DDX3X. **(B)** Exogenous co-IP showing the interaction between Flag-NDFIP2 and Myc-DDX3X. **(C-H)** WT and NDFIP2-KO HCT116 cells were transduced with either shNC or shDDX3X, as indicated. **(C)** Western blot analysis of E-cadherin, N-cadherin, CD133, and OCT4 in WT and NDFIP2 knockout HCT116 cells transduced with shNC or shDDX3X. β-Actin was used as a loading control. **(D, E)** Representative images **(D)** and quantification **(E)** of Transwell migration and invasion assays using WT and NDFIP2 knockout HCT116 cells transduced with shNC or shDDX3X. Scale bars: 200 μm. **(F, G)** Representative images **(F)** and quantification of tumor sphere numbers **(G)** in HCT116 cells following NDFIP2 knockout or DDX3X knockdown. Scale bars: 200 μm. Data in **(E, G)** are presented as mean ± SD (n = 3). Statistical significance was determined using one-way ANOVA followed by Tukey’s multiple-comparisons test. ns, **P < 0.01, ***P < 0.001, and ****P < 0.0001. **(H)** Distribution of tumor spheres according to diameter (50–100 μm, 100–150 μm, and >150 μm) in the indicated groups. **(I)** Representative immunohistochemical staining of DDX3X, p-AKT, and CD133 in T2N0M0 and T2N0M1 CRC tissues. Scale bar: 100 μm. **(J)** Quantification of DDX3X, p-AKT, and CD133 immunohistochemical staining using H-scores in T2N0M0 and T2N0M1 CRC tissues. Data are presented as median with interquartile range (n = 6). Statistical significance was determined using a two-tailed Mann-Whitney U test. **P < 0.01. **(K)** qRT-PCR analysis of DDX3X mRNA expression in vector control and NDFIP2-overexpressing HCT116 cells. Data are presented as mean ± SD (n = 3). Statistical significance was determined using a two-tailed unpaired Student’s t-test. ns, not significant.

**Supplementary Figure S6. NDFIP2 recruits NEDD4 via its PY motifs to promote DDX3X ubiquitination**

**(A)** Exogenous co-IP showing the interaction between Myc-DDX3X and HA-NEDD4. **(B)** Immunofluorescence staining showing the partial colocalization of NDFIP2 and NEDD4. Scale bar: 10 μm. **(C)** Line-scan analysis of NDFIP2 and NEDD4 fluorescence intensity. **(D)** Immunoblot analysis of DDX3X expression in vector- and NEDD4-overexpressing cells. **(E)** Co-IP analysis of the NEDD4–DDX3X interaction in cells with NDFIP2 depletion, NDFIP2 overexpression, or expression of mutant NDFIP2. Data are representative of three independent experiments. IgG served as a negative control for immunoprecipitation assays. β-Actin was used as a loading control where indicated. All experiments shown in panels A-E were performed using HCT116 cells.

**Supplementary Figure S7. GNG13 knockdown suppresses PI3K/AKT signaling and attenuates NDFIP2 loss-induced malignant phenotypes in CRC**

**(A)** Correlation analysis between NDFIP2 and GNG13 expression levels in CRC patients using TCGA database (n = 456). The blue line indicates the linear regression fit with 95% confidence intervals. **(B-D)** qRT-PCR analysis of GNG13 mRNA expression in HCT116 or LoVo cells with DDX3X overexpression or knockdown. **(B, D)** Data are presented as mean ± SD (n = 3). Statistical significance was determined using a two-tailed unpaired Student’s t-test. **P < 0.01. **(C)** Data are presented as mean ± SD (n = 3). Statistical significance was determined using one-way ANOVA followed by Tukey’s multiple comparisons test. ***P < 0.001. **(E)** Immunoblot analysis of NDFIP2, DDX3X, GNG13, PI3K, phospho-PI3K, AKT, and phospho-AKT in HCT116 cells under the indicated conditions. **(F)** Western blot analysis of NDFIP2, GNG13, E-cadherin, N-cadherin, CD133, and OCT4 in WT and NDFIP2 knockout HCT116 cells transfected with control siRNA or siGNG13. β-Actin was used as a loading control. **(G, H)** Representative images **(G)** and quantification **(H)** of Transwell migration and invasion assays using WT and NDFIP2 knockout HCT116 cells transfected with control siRNA or siGNG13. Scale bars: 100 μm. Data are presented as mean ± SD (n = 3). Statistical significance was determined using one-way ANOVA followed by Tukey’s multiple comparisons test. ****P < 0.0001. **(I, J)** Representative images **(I)** and quantification of tumor sphere numbers **(J)** in HCT116 cells following NDFIP2 knockout or GNG13 knockdown. Scale bars: 200 μm. Distribution of tumor spheres according to diameter (50–100 μm, 100–150 μm, and >150 μm) in the indicated groups. Data are presented as mean ± SD (n = 3). Statistical significance was determined using one-way ANOVA followed by Tukey’s multiple comparisons test. **P < 0.01, ****P < 0.0001.

**Supplementary Figure S8. DDX3X resolves promoter-associated R-loops to enhance GNG13 transcription**

**(A)** Dual-luciferase assay showing increased GNG13 promoter activity upon DDX3X overexpression. Data are presented as mean ± SD (n = 3). Statistical significance was determined using a two-tailed unpaired Student’s t-test. **P < 0.01. **(B)** S9.6 dot blot analyses showing reduced R-loop abundance upon DDX3X overexpression, which was abolished by RNase H1 treatment. **(C)** Quantification of nuclear S9.6 fluorescence intensity in shNC and DDX3X-knockdown cells with or without RNase H treatment. Statistical significance was assessed using one-way ANOVA followed by Tukey’s multiple-comparisons test (n = 5). **P < 0.01, ****P < 0.0001. **(D,E)** Representative immunofluorescence images **(D)** and quantification **(E)** of nuclear S9.6 fluorescence intensity in HCT116 cells transfected with either vector or OE-DDX3X, with or without RNase H treatment. Nuclei were counterstained with DAPI. Scale bars: 20 μm. Statistical significance was assessed using one-way ANOVA followed by Tukey’s multiple-comparisons test (n = 5). ****P < 0.0001. **(F, G)** S9.6 DRIP-qPCR analysis of the indicated regions within the GNG13 promoter in HCT116 and LoVo cells, with RNase H treatment as a specificity control. Data are presented as mean ± SD (n = 3). Statistical significance was determined using one-way ANOVA followed by Tukey’s multiple comparisons test. *P < 0.05, ***P < 0.001,****P < 0.0001. **(H, I)** S9.6 DRIP-qPCR showing that DDX3X overexpression reduced R-loop enrichment at the GNG13 promoter in HCT116 and LoVo cells. Data are presented as mean ± SD (n = 3). Statistical significance was determined using two-way ANOVA followed by Šídák’s multiple comparisons test. ***P < 0.001, ****P < 0.0001. **(J)** S9.6 DRIP-qPCR showing that DDX3X knockdown increased R-loop enrichment at the GNG13 promoter in LoVo cells. Data are presented as mean ± SD (n = 3). Statistical significance was determined using one-way ANOVA followed by Tukey’s multiple comparisons test. **P < 0.01, ***P < 0.001, ****P < 0.0001.

**Supplementary Figure S9. DDX3X promotes GNG13 transcription by facilitating CTCF recruitment to its promoter**

**(A, B)** qRT-PCR analysis of GNG13 mRNA expression after CTCF or MYC overexpression in HCT116 and LoVo cells. Data are presented as mean ± SD (n = 3). Statistical significance was determined using one-way ANOVA followed by Tukey’s multiple comparisons test. ns, not significant; ****P < 0.0001. **(C)** Exogenous co-IP assays showing the interaction between DDX3X and CTCF. **(D)** qRT-PCR analysis of CTCF mRNA expression after DDX3X knockdown in LoVo cells. Data are presented as mean ± SD (n = 3). Statistical significance was determined using one-way ANOVA followed by Tukey’s multiple comparisons test. ns, not significant. **(E)** WT and NDFIP2-KO HCT116 cells were transduced with either shNC or shDDX3X, as indicated. Immunoblot analysis of CTCF protein expression in WT, NDFIP2-knockout, and DDX3X-knockdown HCT116 cells. **(F-G)** NDFIP2 and RNASEH1 modulate cell motility. **(F)** Representative images of Transwell migration and invasion assays in LoVo cells overexpressing NDFIP2 with or without RNASEH1. Scale bar: 200 μm. **(G)** Quantitative analysis of migrated and invaded cells per field. Data are presented as mean ± SD (n = 3). Statistical significance was determined using one-way ANOVA followed by Tukey’s multiple comparisons test. *P < 0.05,**P < 0.01, ***P < 0.001,****P < 0.0001.

**Supplementary Figure S10. The iRGD-conjugated NDFIP2 residues 147-220 peptide inhibits CRC metastatic progression *in vitro* and *in vivo***

**(A, B)** Representative Transwell images and quantification showing that ND-iRGD reduced the migratory and invasive capacities of LoVo cells. Scale bar: 200 μm. Data are presented as mean ± SD (n = 3). Statistical significance was determined using a two-tailed unpaired Student’s t-test. **P < 0.01, ***P < 0.001. **(C)** Quantification of tumorsphere formation showing that ND-iRGD decreased the number of tumor spheres in HCT116 and LoVo cells. Data are presented as mean ± SD (n = 3). Statistical significance was determined using a two-tailed unpaired Student’s t-test. **P < 0.01. **(D)** Immunoblot analysis of stemness-associated markers in cells treated with iRGD or ND-iRGD. **(E)** Quantification of immunohistochemical staining for DDX3X, p-AKT, E-cadherin, N-cadherin, and CD133 in tumor tissues from the iRGD and ND-iRGD groups using H-scores (n = 5). Data are presented as median with interquartile range (n = 5 mice per group). Statistical significance was determined using a two-tailed Mann-Whitney U test. *P < 0.05, **P < 0.01. **(F)** Representative bioluminescence images and quantification of liver metastases acquired by IVIS three weeks after tumor cell inoculation in mice treated with iRGD or ND-iRGD (n = 5). Beginning 1 week after tumor cell inoculation, mice received intraperitoneal injections of the indicated peptides at 25 μmol/kg every 3 days for 2 weeks. **(G)** Quantification of the number of metastatic liver nodules in each group. Data are presented as mean ± SD (n = 5). Statistical significance was determined using one-way ANOVA followed by Tukey's multiple comparisons test. ns, not significant; *P < 0.05. **(H)** Representative hematoxylin and eosin (H&E) staining of liver sections from mice in each group. Arrows indicate metastatic lesions. Scale bar: 500 μm. **(I)** Representative immunohistochemical staining of DDX3X, p-AKT, E-cadherin, N-cadherin, and CD133 in liver metastatic lesions from the indicated groups. Scale bar: 100 μm. **(J)** Quantification of immunohistochemical staining for DDX3X, p-AKT, E-cadherin, N-cadherin, and CD133 in tumor tissues from the iRGD and ND-iRGD groups using H-scores (n = 5). Data are presented as median with interquartile range (n = 5 mice per group). Statistical significance was determined using a two-tailed Mann-Whitney U test. **P < 0.01. **(K)** Kaplan‒Meier analysis showing the survival period of the mice in each group (n = 5). Statistical significance was determined using the log-rank (Mantel–Cox) test. P < 0.05 was considered statistically significant. For panels A-D, CRC cells were treated with the ND-iRGD fusion peptide or the iRGD control peptide at 10 μM for 24 h before the indicated assays.

**Supplementary Figure S11. GNG13 overexpression counteracts the inhibitory effects of ND-iRGD on CRC progression**

**(A,B)** Western blot analysis of E-cadherin, N-cadherin, p-PI3K, p-AKT, CD133, and OCT4 in HCT116 cells transfected with the vector or the GNG13 overexpression construct and treated with iRGD or ND-iRGD at 10 μM for 24 h. β-Actin was used as a loading control. **(C,D)** Representative images **(C)** and quantification **(D)** of Transwell migration and invasion assays performed using the indicated HCT116 cells after treatment with iRGD or ND-iRGD at 10 μM for 24 h. Scale bars: 200 μm. Data are presented as mean ± SD (n = 3). Statistical significance was determined using one-way ANOVA followed by Tukey’s multiple comparisons test. ****P < 0.0001. **(E,F)** Representative images **(E)**, total numbers, and size distributions **(F)** of tumorspheres formed by HCT116 cells transfected with the vector or the GNG13 overexpression construct. Cells were treated with iRGD or ND-iRGD at 10 μM beginning on day 4, and tumorspheres were imaged and quantified on day 7. Tumorspheres were classified according to diameter as 50-100, 100-150, or >150 μm. Scale bars: 200 μm. Data are presented as mean ± SD (n = 3). Statistical significance for tumorsphere numbers was determined using one-way ANOVA followed by Tukey’s multiple comparisons test. ns, not significant; **P < 0.01.
